# Division of labor of Y-family polymerases in translesion-DNA synthesis for distinct types of DNA damage

**DOI:** 10.1371/journal.pone.0252587

**Published:** 2021-06-01

**Authors:** Yuriko Inomata, Takuya Abe, Masataka Tsuda, Shunichi Takeda, Kouji Hirota

**Affiliations:** 1 Department of Chemistry, Graduate School of Science, Tokyo Metropolitan University, Hachioji-shi, Tokyo, Japan; 2 Department of Radiation Genetics, Graduate School of Medicine, Kyoto University, Sakyo-ku, Kyoto, Japan; 3 Program of Mathematical and Life Sciences, Graduate School of Integrated Sciences for Life, Hiroshima University, Higashi-Hiroshima, Japan; Institut Pasteur, FRANCE

## Abstract

Living organisms are continuously under threat from a vast array of DNA-damaging agents, which impact genome DNA. DNA replication machinery stalls at damaged template DNA. The stalled replication fork is restarted via bypass replication by translesion DNA-synthesis polymerases, including the Y-family polymerases Polη, Polι, and Polκ, which possess the ability to incorporate nucleotides opposite the damaged template. To investigate the division of labor among these polymerases *in vivo*, we generated *POLη*^*−/−*^, *POLι*^*−/−*^, *POLκ*^*−/−*^, double knockout (KO), and triple knockout (TKO) mutants in all combinations from human TK6 cells. TKO cells exhibited a hypersensitivity to ultraviolet (UV), cisplatin (CDDP), and methyl methanesulfonate (MMS), confirming the pivotal role played by these polymerases in bypass replication of damaged template DNA. *POLη*^*−/−*^ cells, but not *POLι*^*−/−*^ or *POLκ*^*−/−*^ cells, showed a strong sensitivity to UV and CDDP, while TKO cells showed a slightly higher sensitivity to UV and CDDP than did *POLη*^*−/−*^ cells. On the other hand, TKO cells, but not all single KO cells, exhibited a significantly higher sensitivity to MMS than did *wild-type* cells. Consistently, DNA-fiber assay revealed that Polη plays a crucial role in bypassing lesions caused by UV-mimetic agent 4-nitroquinoline-1-oxide and CDDP, while all three polymerases play complementary roles in bypassing MMS-induced damage. Our findings indicate that the three Y-family polymerases play distinctly different roles in bypass replication, according to the type of DNA damage generated on the template strand.

## Introduction

Genomic DNA, the genetic blueprint of life, is replicated with remarkably high fidelity by replicative polymerases Polα, Polδ, and Polε to precisely maintain genetic information [1–4]. The DNA replication fork is frequently stalled by damages induced not only by external factors (such as ultraviolet [UV] light from sunlight and environmental chemicals) but also by internal factors (such as oxygen radicals and aldehyde resulting from metabolic reactions). Stalled replication forks are restarted by DNA-damage tolerance (DDT) pathways that bypass DNA damage without repairing the lesions, allowing replication to resume beyond those lesions. In eukaryotic cells, there are two distinct DDT mechanisms: homologous recombination (HR) and translesion DNA synthesis (TLS) [5]. HR mediates continuous replication by switching from the damaged template strand to the newly synthesized sister strand [5–9], while TLS mediates reversible switching between replicative polymerases and specialized TLS polymerases to directly bypass the damaged template [10–13].

TLS polymerases are best characterized by the Y-family DNA polymerases, encompassing Polη, Polι, and Polκ [14–16]. These three polymerases share structural similarities and play roles in the insertion of nucleotides opposite the damaged template. Polη mutations have been identified as being responsible for the variant form of xeroderma pigmentosum (XP-V), which is characterized by UV photosensitivity and predisposition to skin cancer [17]. This polymerase is conserved from yeast (Rad30) to humans [17]; it exhibits enzymatic properties to efficiently and correctly replicate DNA past a UV-induced cyclobutane pyrimidine dimer (CPD) in template DNA [18]. Polι, originally named Rad30B, was discovered as the second human ortholog of yeast Rad30; its ability to replicate by bypassing the damaged template has been confirmed [19, 20]. Polι physically interacts with Polη, with the localization of these two polymerases being tightly coordinated within the nucleus [21]. However, the function of Polι remains uncertain, since *POLI*^*−/−*^ and *wild-type* mice show a very similar phenotype in all aspects of growth and mutation rates [22, 23]. We know that the third Y-family DNA polymerase, Polκ, is specialized to bypass bulky DNA adducts, such as benzo[*a*]pyrene, *in vitro* [24, 25]. Yet the full function of Polκ also remains uncertain, since its inactivation has little impact on mutagenesis induced by benzo[*a*]pyrene [26]. Redundancy of three Y-family DNA polymerases in cellular response to DNA damages induced by UV was demonstrated in mouse embryonic fibroblast cells [27]. Similarly, involvement of Polι in the TLS of UV-induced damage as a backup for Polη was reported in human Burkitt’s lymphoma BL2 cell line [28]. Moreover, mouse embryonic fibroblast cells defective for all three Y-family DNA polymerases exhibit highest sensitivities to wide variety of DNA damaging agents including methyl methanesulfonate (MMS) in comparison to each single mutant cells [29]. These studies had suggested redundant role of these Y-family DNA polymerases in mouse. However, the functional relationship among Y-family DNA polymerases in human cell has not been fully elucidated due to the absence of triple- knockout (TKO) human cell line.

In this study, we investigated the relationship and the division of labor in the three Y-family polymerases (Polη, Polι, and Polκ) by generating *POLH*^*−/−*^, *POLI*^*−/−*^, *POLK*^*−/−*^, double knockout (KO), and TKO mutants in all combinations from human TK6 cells. We found that the relationship among the three Y-family polymerases distinctly differs according to the type of DNA damage generated on the template-strand DNA.

## Materials and methods

### TK6 cell culture

TK6 cells [30] were cultured in Roswell Park Memorial Institute 1640 medium (Nacalai Tesque, Kyoto, Japan) supplemented with 10% heat-inactivated horse serum from Gibco and sodium pyruvate (0.1 mM), L-glutamine (2 mM), penicillin (100 U/mL), and streptomycin (100 μg/mL) from Nacalai Tesque. TK6 cells have a stable, near-diploid karyotype, except for a trisomic chromosome 13 [31].

### Plasmids

We used pX330 vector [32] (Addgene, US) for the CRISPR-Cas9 system. The marker genes used in this study were *DT-ApA*/*NEO*^*R*^ (provided by the Laboratory for Animal Resources and Genetic Engineering, Center for Developmental Biology, RIKEN Kobe), *DT-ApA*/*PURO*^*R*^, *DT-ApA*/*BSR*^*R*^, *DT-ApA*/*HIS*^*R*^, *DT-ApA*/*HYG*^*R*^, and *DT-ApA*/*ZEO*^*R*^.

### Genotoxic reagents

Olaparib (AZD-2281, AstraZeneca), ICRF193, MMS (Nacalai Tesque, Japan), camptothecin (CPT; Topogen), cisplatin (CDDP; Nihonkayaku), and 4-nitroquinoline 1-oxide (4NQO) were used as described [33–35].

### Antibodies

anti-Polη antibody (A301-231A, Bethyl, TX), anti-Polι antibody (A301-304A, Bethyl, TX), and anti-Polκ antibody (A301-977A,Bethyl, TX) were used for western blot analysis.

### Measurement of cellular sensitivity to DNA-damaging agents

To measure cellular sensitivity to olaparib, ICRF193, MMS, CPT, and CDDP, a liquid-culture cell-survival assay was employed as previously described [36, 37]. Briefly, TK6 cells were diluted in medium (10^4^ cells/ml) and dispensed into a 24-well plate (1 ml) to which the above-mentioned DNA-damaging agents were added and mixed before culturing for 72 h. To measure sensitivity to γ- and UV-rays, irradiated cells were transferred to the culture medium (1 ml) and cultured for 72 h at 37° C. The incubated cells (100 μl) were then transferred to 96-well plates, and the amount of ATP was measured using the CellTiter-Glo Cell Viability Assay (Promega), according to the manufacturer’s instructions. Luminescence was measured using a Fluoroskan Ascent FL Microplate Fluorometer and Luminometer (Thermo Fisher Scientific Inc., Waltham, MA). Sensitivity to UV was also verified by conventional colony survival assay. Briefly, TK6 cells were suspended in phosphate-buffered saline containing 1% heat-inactivated horse serum and exposed to UV-ray (λ = 260 nm) and seeded in drug-free medium containing 1.5% methylcellulose, and then cultured at 37°C for 14 day.

### Generation of *POLH*^*−/−*^, *POLI*^*−/−*^, and *POLK*^*−/−*^ mutant TK6 cells

*POLH* was disrupted, as previously described [38].

*POLI* was disrupted with KO constructs prepared using primers 5′-GCGAATTGGGTACCGGGCCAGGGATTTGTCCTGTGACCTAAAATCAGTC-3′ and 5′-CTGGGCTCGAGGGGGGGCCTCTCTCAACAACTGGACTAAATTCTTCCAG-3′ for the left arm and 5′-TGGGAAGCTTGTCGACTTAAGTCATGTATACAATAATCAGTGTGAGTGGG-3′ and 5′-CACTAGTAGGCGCGCCTTAAAACTATTCTGTAGACCGATGTCTAGTTCTC-3′ for the right arm. The PCR-amplified left and right arms were inserted in marker-gene plasmids (above described *DT-ApA*/*NEO*^*R*^ based plasmids) digested with *Apa*I and *Afl*II using the GeneArt Seamless Cloning & Gibson Assembly system (ThermoFisher, PA) to KO construct. The resultant KO plasmids express diphtheria toxin from outside of the homologous arms to suppress random integration event. The CRISPR expression vector for the CRISPR-Cas9 system was designed to recognize 5′-ACTTTCTGCGGTGACTGTGT-3′ (S1 Fig).

*POLK* was disrupted with KO constructs prepared using primers 5′-GCGAATTGGGTACCGGGCCAACATGAGTCAGGGTGATTGCTTCTAAAAG-3′ and 5′-CTGGGCTCGAGGGGGGGCCTGTTCTAATTCCATTGCAAATCTGTCAACC-3′ for the left arm and 5′-TGGGAAGCTTGTCGACTTAACCATTGCTGTAGGATCAATGAGTATGCTGG-3′ and 5′-CACTAGTAGGCGCGCCTTAAGCAGTTGGTTTAGCTTTAACATGGCTACAG-3′ for the right arm. The PCR-amplified left and right arms were inserted in marker-gene plasmids digested with *Apa*I and *Afl*II using the GeneArt Seamless Cloning & Gibson Assembly system (ThermoFisher, PA). The CRISPR expression vector for the CRISPR-Cas9 system was designed to recognize 5′-CACCATCCATGTCAATGTGCACTA-3′ (S1 Fig).

*Wild-type* TK6 cells were transfected with the above-mentioned targeting vectors (2 μg) and the expression vector (7 μg) for CRISPR using the NEON Transfection System (Invitrogen, CA) at 1400 V with a pulse width of 20 ms. The loss of the *POLI* or *POLK* transcript was confirmed by RT-PCR using primers 5′-GTTAATGGAGAAGACCTGACCCGCTACAG 3′ and 5′-GCAGAAGCACACACTTAGAAGGGTAAGG -3′, and 5′-ATGGATAGCACAAAGGAGAAGTGTGAC -3′ and 5′-CTATTATAAGTTGTGGGCACAGCCTC-3′, respectively. β-actin transcript was used as a positive control for the RT-PCR analysis using primers 5′-GATGGTGGGCATGGGTCAGAAGGATTCC-3′ and 5′-GTCCAGGGCGACGTAGCACAGCTTCTC-3′. The MIT specificity scores for each gRNA were calculated according to the method by Haeussler et al [39].

### Chromosomal aberration analysis

TK6 cells were treated with colcemid (Gibco BRL) (0.1 μg/ml) for 3 h to arrest them in the M phase. The cells were then pelleted by centrifugation, resuspended in 75 mM KCl (10 ml) for 13 min at room temperature, and fixed in a freshly prepared 3:1 mixture of methanol and acetic acid (Carnoy’s solution) (2 ml). The pelleted cells were then resuspended in Carnoy’s solution (7 ml), dropped onto cold glass slides, and air-dried. The slides were stained with a 5% HARLECO Giemsa Stain solution (Nacalai Tesque) for 10 min, rinsed with water and acetone, and dried at room temperature. All chromosomes in each mitotic cell were scored at 1000× magnification.

### Measurement of sister-chromatid exchanges (SCEs)

SCEs were measured as previously described, with slight modifications [40]. Briefly, TK6 cells were incubated with CDDP (0.2 μM) or MMS (1 μg/mL) and with bromodeoxyuridine (BrdU; 10 μM) for 24 h, which corresponds to two cell-cycle periods for ΤΚ6 cells. For UV irradiation, cells were exposed to UV (1 J/m^2^), then cultured in a medium containing BrdU for 24 h. The cells were treated with colcemid (0.1 μg/ml) to enrich for mitotic cells for the last 3 h of incubation. The cells were then pelleted by centrifugation, resuspended in 75 mM KCl (0.2 ml) for 13 min at room temperature, and fixed in freshly prepared Carnoy’s solution (0.4 ml). The pelleted cells were then resuspended in Carnoy’s solution (0.4 ml), after which the solution was dropped onto clean glass slides and air-dried. The dried slides were incubated with Hoechst 33258 nuclei acid stain (10 μg/ml) in phosphate buffer (pH 6.8) for 20 min and rinsed with McIlvaine buffer (164 mM Na_2_HPO_4_ and 16 mM citric acid [pH 7.0]). Next, the slides were irradiated with black light (λ = 352 nm) for 25 min and incubated in a saline-sodium citrate (0.15 M NaCl plus 0.015 M sodium citrate) solution at 58° C for 20 min, after which they were stained with 5% HARLECO Giemsa Stain Solution (Nacalai Tesque) for 10 min. We scored 50 Giemsa-stained metaphase cells per test at 1000× magnification.

### DNA-fiber assay

The DNA-fiber assay was performed as previously described [36, 37], with a slight modification in the labeling method for the replicated tract. Briefly, cells were sequentially labeled for 15 min each with 25 μM 5-chloro-2’-deoxyuridine (CldU) and 250 μM 5-iodo-2’-deoxyuridine (IdU). The fiber length was measured using Image J software (https://imagej.nih.gov/ij/), and the CldU/IdU ratio was calculated. Measurements were recorded from areas of the slides with untangled DNA fibers to prevent the possibility of recording labeled patches from tangled bundles of fibers.

## Results

### Y-family DNA polymerases Polη, Polι, and Polκ are required for cellular tolerance to UV, CDDP, and MMS

To examine the functional relationship among Polη, Polι, and Polκ *in vivo*, we generated *POLH*^*−/−*^, *POLI*^*−/−*^, *POLK*^*−/−*^, double KO, and TKO mutants in all combinations from human TK6 cells (S1 Fig). The loss of mRNA and protein expressions was verified by RT-PCR and western blot analysis, respectively (S2 Fig). *POLH*^*−/−*^, *POLI*^*−/−*^, *POLK*^*−/−*^, and *POLH*^*−/−*^*/POLI*^*−/−*^*/POLK*^*−/−*^ (TKO) cells proliferated with normal kinetics (S3A Fig) and exhibited similar cell-cycle distribution, compared with *wild-type* cells (S3B Fig). These results suggest that the three Y-family polymerases are dispensable for unperturbed replication.

**Fig 1:**
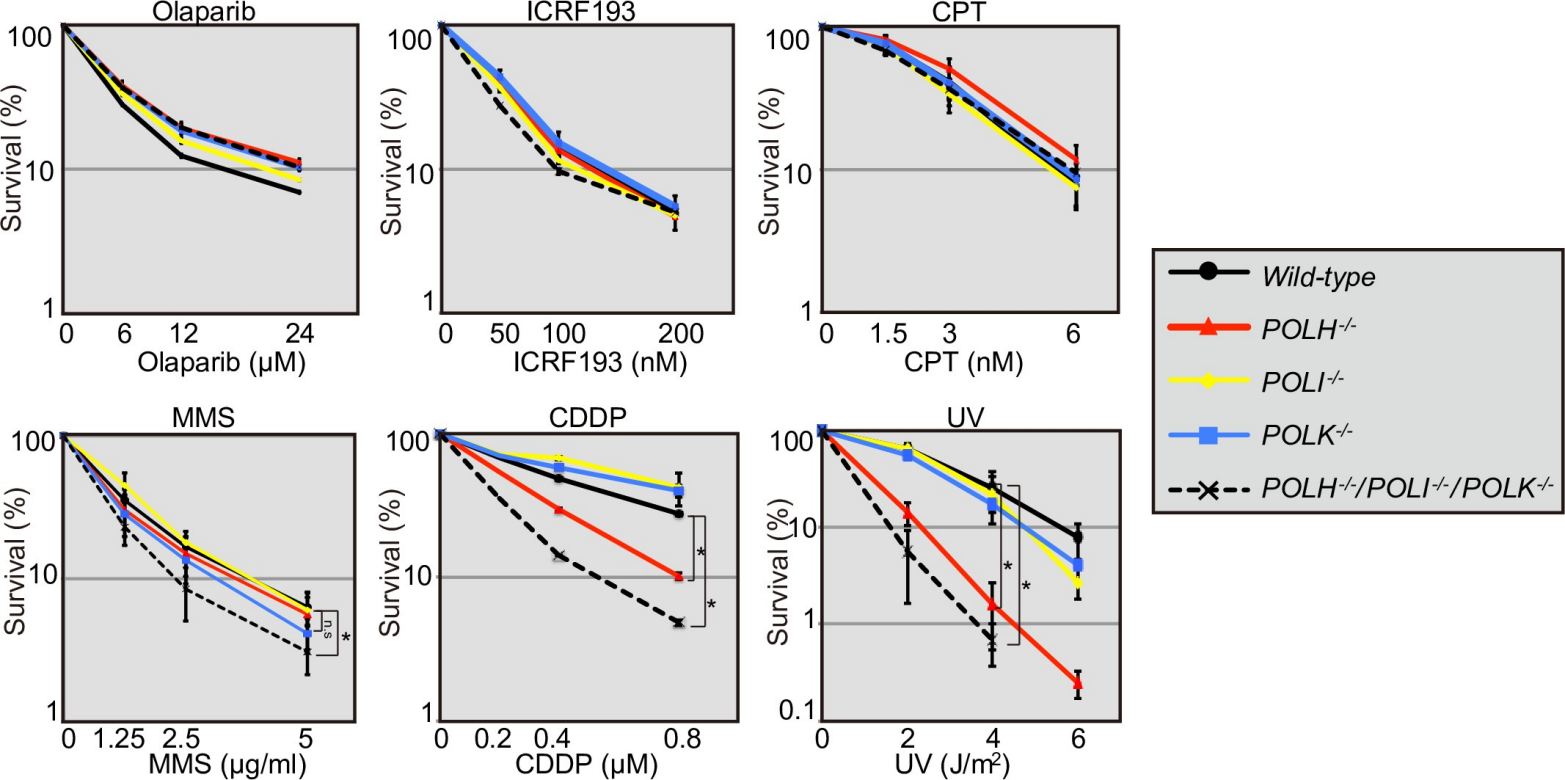
Role of Y-family polymerases in cellular tolerance to DNA-damaging agents. TK6 cells were assessed for sensitivity to six DNA-damaging agents. Cell viability was assessed by ATP assay, as described in the Materials and Methods. The dose of the indicated DNA-damaging agent is displayed on the x-axis on a linear scale, while the percentage of cell survival is displayed on the y-axis on a logarithmic scale. Error bars represent the standard deviation from three independent measurements. The *p*-value was calculated by Student’s *t*-test (*p < 0.05 and n.s [not significant]).

In order to explore the various roles played by these polymerases in genome maintenance, we measured cellular sensitivity to a broad range of DNA-damaging agents. TKO cells were not sensitive to olaparib, ICRF193, or CPT (Fig 1). Olaparib inhibits enzymatic activity of PARP and induces single strand breaks associated with PARP trapping, which in turn converts in DNA double strand breaks during DNA synthesis [33]. CPT and ICR193 are inhibitor of topoisomerase I and II, respectively, and induce DNA strand breaks repaired by HR and non-homologous end-joining, respectively [41]. Similar sensitivity between TKO and *wild-type* cells to olaparib, ICRF193, and CPT suggests that these polymerases are not vital for strand-break repair (Fig 1). By contrast, TKO cells were more sensitive to UV, CDDP, and MMS than were the *wild-type* cells (Fig 1). For measuring cellular sensitivity, we employed liquid-culture cell-survival assay, since validity of this assay has been confirmed for several DNA damaging agents including UV and γ-ray [42]. Indeed, conventional colony survival assay showed that TKO cells are more sensitive to UV than are the *wild-type* cells (S4 Fig). Moreover, the other TKO clones also showed similarly higher sensitivity to UV, CDDP, and MMS than did *wild-type* cell, and each independent TKO clones surely showed similar sensitivity (S5 Fig), indicating that the hypersensitivity shown in TKO cells might not be caused by clonal variations. Hypersensitivity to a wide range of DNA-replication-blocking agents has also been observed in TLS-deficient mutants, such as *RAD18*^*−/−*^, *REV3*^*−/−*^, and *PCNA*^*-/K164R*^ cells [43–45], confirming that these Y-family polymerases are indeed involved in the bypass replication of damaged templates DNA [25] (Fig 1). Moreover, several former studies have shown the UV-hypersensitivity of Polη deficient cells but some other reports have not supported these results, and thus the contribution of Polη in UV-tolerance has been controversial. The data showing UV-hypersensitivity in *POLH*^-/-^ cells might contribute to the resolution of this discrepancy (Fig 1).

### Polη, Polι, and Polκ play complementary roles in cellular tolerance to MMS

**Fig 2:**
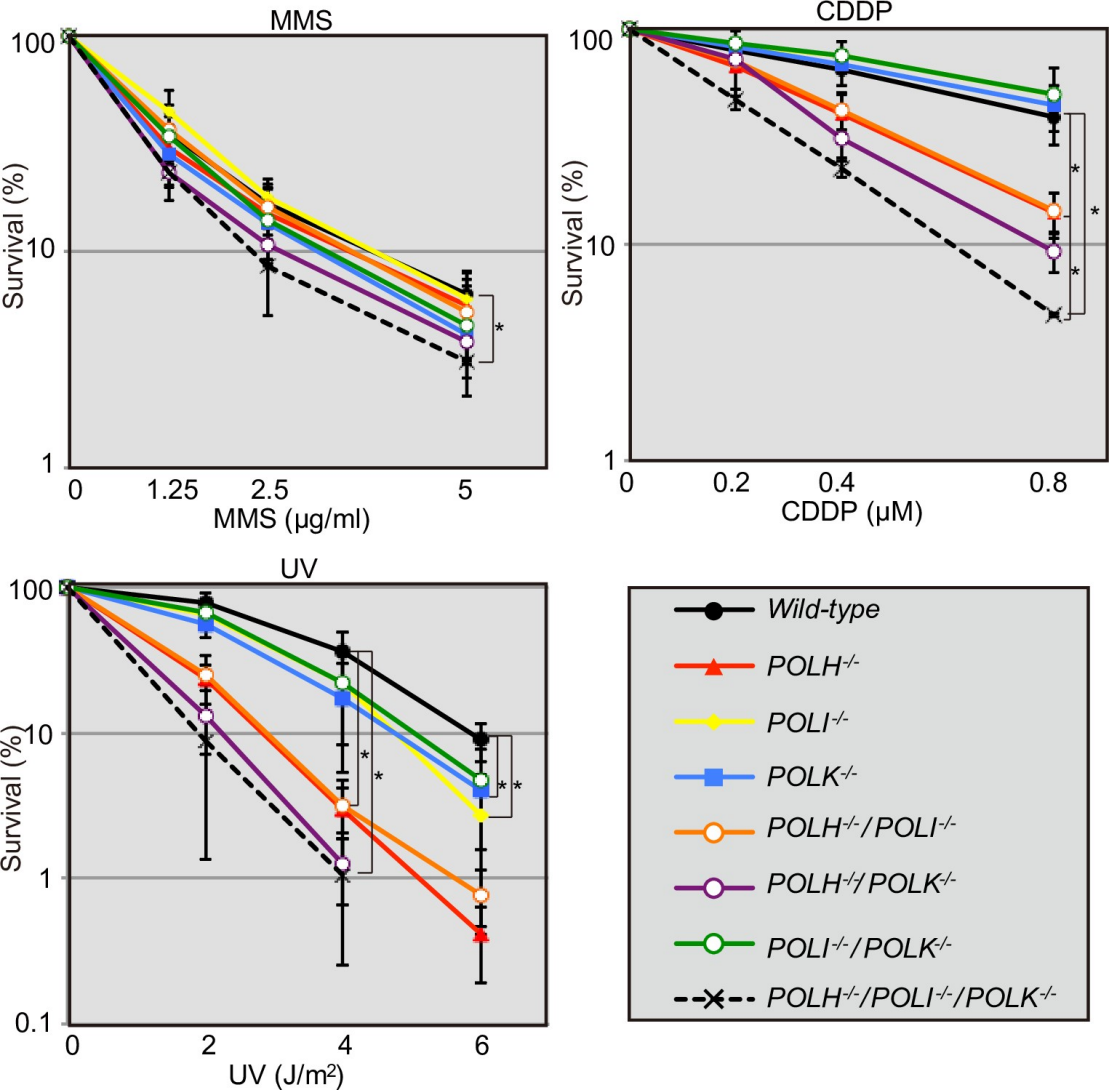
The relationship between Polη, Polι, and Polκ in cellular tolerance to MMS, CDDP, and UV. (A-C) Indicated TK6 cells were assessed for sensitivity to MMS (A), CDDP (B), and UV (C) as in Fig 1. Data for the CDDP sensitivity for *POLI*^*−/−*^ were completely overlayed by the data for *POLI*^*−/−*^/*POLK*^*−/−*^. The dose of DNA-damaging agents is displayed on the x-axis on a linear scale, while the percentage of cell survival is displayed on the y-axis on a logarithmic scale. Error bars represent the standard deviation from three independent experiments. The *p*-value was calculated by Student’s *t*-test (*p < 0.05).

**Fig 3:**
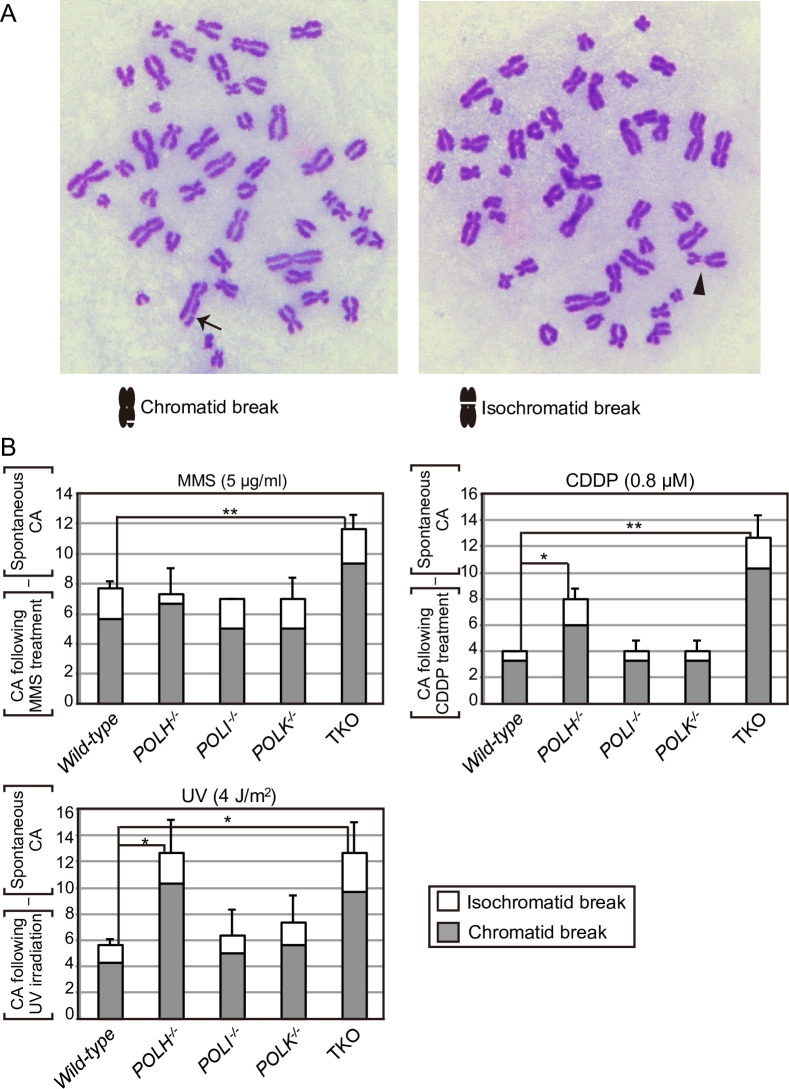
Contribution of Polη, Polι, and Polκ in preventing chromosomal breakage after exposure to MMS, CDDP, or UV. (A) Representative images showing chromosomal aberrations. The arrow and arrowhead indicate a chromatid break and an isochromatid break, respectively. (B) TK6 cells were cultured in a medium containing CDDP (0.8 μM) or MMS (5 μg/mL) for 12 h or exposed to UV (4 J/m^2^) and cultured for 12 h. Cells were treated with colcemid for the last 3 h. The number of chromosomal aberrations (CAs) per 100 mitotic cells before and after the 12-h treatment was scored three times; the average and standard deviation from three experiments are presented in S6 Fig. The number of spontaneous CAs was subtracted from the number of DNA-damaging agent-induced CAs and is shown in the histogram. Error bars represent the standard deviation from three independent measurements. The *p*-value was calculated by Student’s *t*-test (*p < 0.05, ** *p* < 0.01).

To further investigate the roles of the three Y-family DNA polymerases in cellular tolerance to the DNA replication blocking agents, MMS, CDDP, and UV, we examined cellular tolerance in *POLH*^*−/−*^, *POLI*^*−/−*^, *POLK*^*−/−*^, double KO, and TKO mutants in all combinations (Fig 2). MMS induces base methylation, thereby interfering with the progression of replicative polymerases [36, 37]. *POLH*^*−/−*^, *POLI*^*−/−*^, and *POLK*^*−/−*^ cells were not sensitive to MMS, whereas TKO cells showed a significantly higher sensitivity to MMS than did *wild-type* cells (Figs 1 and 2 *p<0.05). TKO cells consistently showed increased chromosomal aberrations after MMS treatment (Fig 3 *p<0.05, S6 Fig). These data indicate that the three Y-polymerases act complementarily for bypass replication across templates carrying MMS-induced methylated bases.

### Polη plays major roles in cellular tolerance to CDDP

CDDP induces intra-strand crosslinks and inter-strand crosslinks (ICLs); replication is stalled in both instances. ICLs are the most formidable type of DNA-damage induced by CDDP, since the repair of ICLs at the replication fork is complex and involves multiple repair processes, including TLS [46, 47]. *POLI*^*−/−*^ and *POLK*^*−/−*^ cells were not sensitive to CDDP (Figs 1 and 2). By contrast, *POLH*^*−/−*^ cells showed a markedly higher sensitivity to CDDP than did *wild-type* cells (Fig 1 *p<0.05, Fig 2). The loss of Polκ in *POLH*^*−/−*^ cells slightly enhanced their sensitivity to CDDP. The loss of both Polι and Polκ in *POLH*^*−/−*^ cells further increased that sensitivity and TKO showed a significantly higher sensitivity to CDDP (Figs 1 and 2 *p<0.05). *POLH*^*−/−*^ and TKO consistently showed increased levels of chromosomal aberrations after CDDP treatment (Fig 3 *p<0.05, S6 Fig). These results suggest that Polη plays a major role in the bypass replication of DNA lesions induced by CDDP, including ICLs, and that other polymerases served as a backup for Polη.

### Dominant contribution of Polη-Polι to cellular tolerance to UV

We next examined the relationships among the three Y-family DNA polymerases in cellular tolerance to UV. *POLI*^*−/−*^ and *POLK*^*−/−*^ cells exhibited moderate sensitivity to UV than did *wild-type* cells (Figs 1 and 2), suggesting that Polι and Polκ have some role(s) in the bypass replication beyond UV-induced lesions. On the other hand, *POLH*^*−/−*^ cells showed significantly higher sensitivity to UV than did *wild-type*, *POLI*^*−/−*^, and *POLK*^*−/−*^ cells (Figs 1 and 2 *p<0.05). These results indicate the prominent role played by Polη in bypass replication beyond UV-induced lesions. Loss of Polι had no effect on cellular sensitivity to UV in *POLH*^*−/−*^ or *POLK*^*−/−*^ cells (Fig 2), indicating that Polι cannot contribute to the cellular tolerance to UV in the absence of Polη or Polκ. These data suggest the collaborative action of Polη-Polι and Polκ-Polι in cellular tolerance to UV. Since the UV sensitivity of *POLI*^*−/−*^/*POLK*^*−/−*^ cells was critically weaker than that of the *POLH*^*−/−*^/*POLI*^*−/−*^ cells, Polκ-Polι’s role in cellular UV tolerance might not be vital, if it exists at all. Moreover, the loss of Polκ in *POLH*^*−/−*^ or *POLH*^*−/−*^/*POLI*^*−/−*^ cells further reduced cellular viability in response to UV treatment (Figs 1 and 2 *p<0.05), suggesting that Polκ acts as a backup for Polη-Polι-mediated TLS. These relationships were also verified by the conventional colony survival assay (S4 Fig). Moreover, *POLH*^*−/−*^ and TKO cells consistently showed an increase in chromosomal aberrations after UV treatment (Fig 3, S6 Fig *p<0.05). These results suggest that Polη and Polι collaborate to play a dominant role in the bypass replication of DNA lesions induced by UV.

### Polη, Polι, and Polκ play complementary roles in the maintenance of fork progression after MMS-mediated base damage

**Fig 4:**
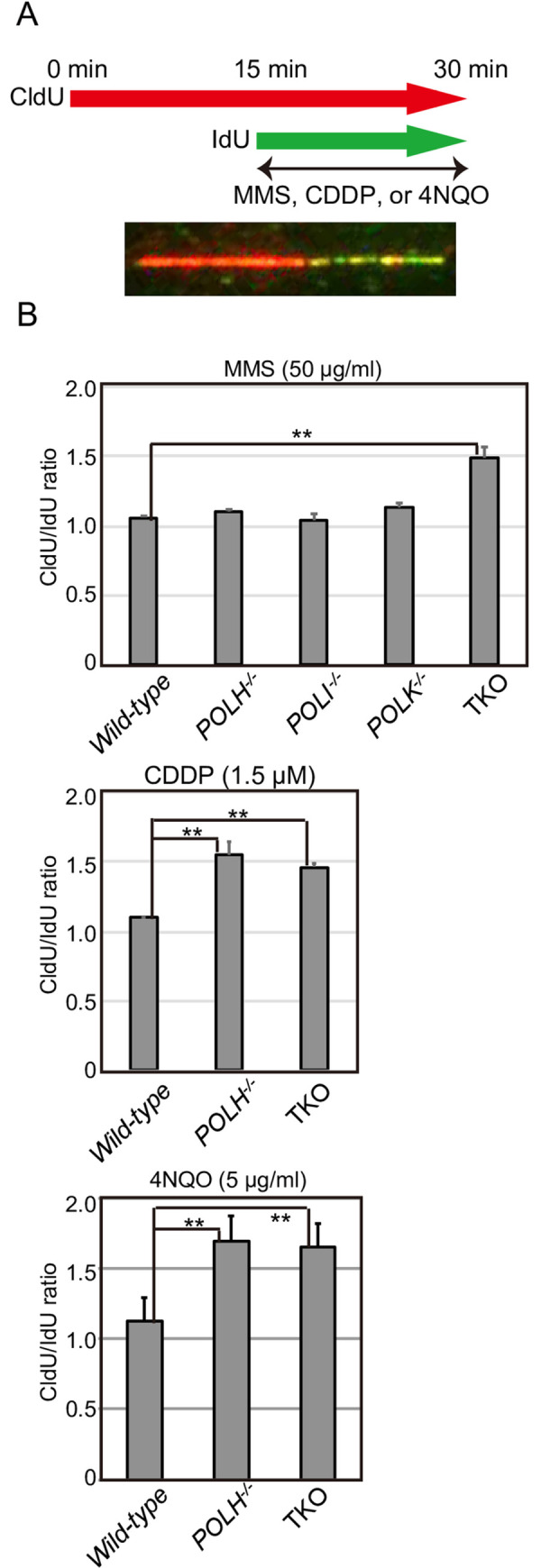
Relationship between Polη, Polι, and Polκ in replication-fork progression after exposure to MMS-, CDDP-, or 4NQO-damaged DNA. (A) Representative image showing stained DNA fibers. TK6 cells were labeled sequentially with CldU and IdU and treated with MMS (50 μg/mL), CDDP (1.5 μM), or 4NQO (5 μg/mL) after CldU labeling. (B) The lengths of the CldU and IdU tracts were measured, and the CldU/IdU ratio for each replication fork was calculated for at least 100 replication forks. The assay was carried out independently 2 times. The averages and standard deviations of medians are shown in the histograms. The *p*-value was calculated by Student’s *t*-test (**p < 0.01). Each data set is shown in S7 Fig.

The hypersensitivity to MMS found in TKO cells, but not in any of the single KO cells, suggests that the three Y-family polymerases complementarily participate in bypass replication across base damage induced by MMS. To directly investigate fork progression, we employed a DNA-fiber assay, using a method for labeling newly synthesized tracts *in vivo* [48]. We sequentially pulse-labeled nascent strands with CldU and IdU for 15 min each, treated the cells with MMS (50 μg/ml) during the second pulse-labeling (Fig 4A). We then measured the length of the replicated tract before (CldU, red) and after (IdU, green) MMS treatment and calculated the ratios to compare total DNA synthesis before and after MMS treatment (Fig 4B). *Wild-type* and all types of single KO cells showed a similar length of DNA synthesis before and after MMS treatment, with a median CldU/IdU ratio of 1–1.13. We detected a definite shortening of IdU-labeling (after MMS treatment) in TKO cells, with a median CldU/IdU ratio of 1.49 ± 0.08 (Fig 4B). These data indicate that the three Y-family polymerases play complementary roles in TLS across MMS-induced DNA lesions.

### Polη plays a major role in the bypass replication of lesions induced by CDDP and the UV-mimetic agent 4NQO

We examined replication-fork progression by DNA-fiber assay after treatment with UV-mimetic agent 4NQO or CDDP. Since only *POLH*^*−/−*^, but not *POLI*^*−/−*^ or *POLK*^*−/−*^ cells, exhibited a markedly higher sensitivity and an increase in chromosomal aberrations after CDDP or UV treatment (Figs 1–3), we measured DNA replication in *wild-type*, *POLH*^*−/−*^, and TKO cells before and after CDDP or 4NQO treatment. *Wild-type* cells showed a similar length of DNA synthesis before and after CDDP or 4NQO treatment (Fig 4). Unlike *wild-type* cells, *POLH*^*−/−*^ cells exhibited a definite shortening of IdU-labeling after CDDP or 4NQO treatment, with median CldU/IdU ratios of 1.55 ± 0.10 and 1.69 ± 0.16, respectively (Fig 4). Interestingly, the CldU/IdU ratio of the *POLH*^*−/−*^ cells after CDDP or 4NQO treatment was indistinguishable from that of the TKO cells. It is thus possible that the higher sensitivity to CDDP or UV in the TKO cells, compared with the *POLH*^*−/−*^ cells, is attributable to the contribution of Polκ to nucleotide excision repair [49]. In summary, these data indicate that Polη plays a major role in the bypass replication of DNA lesions induced by CDDP or UV.

### SCEs were increased by TLS failure

**Fig 5:**
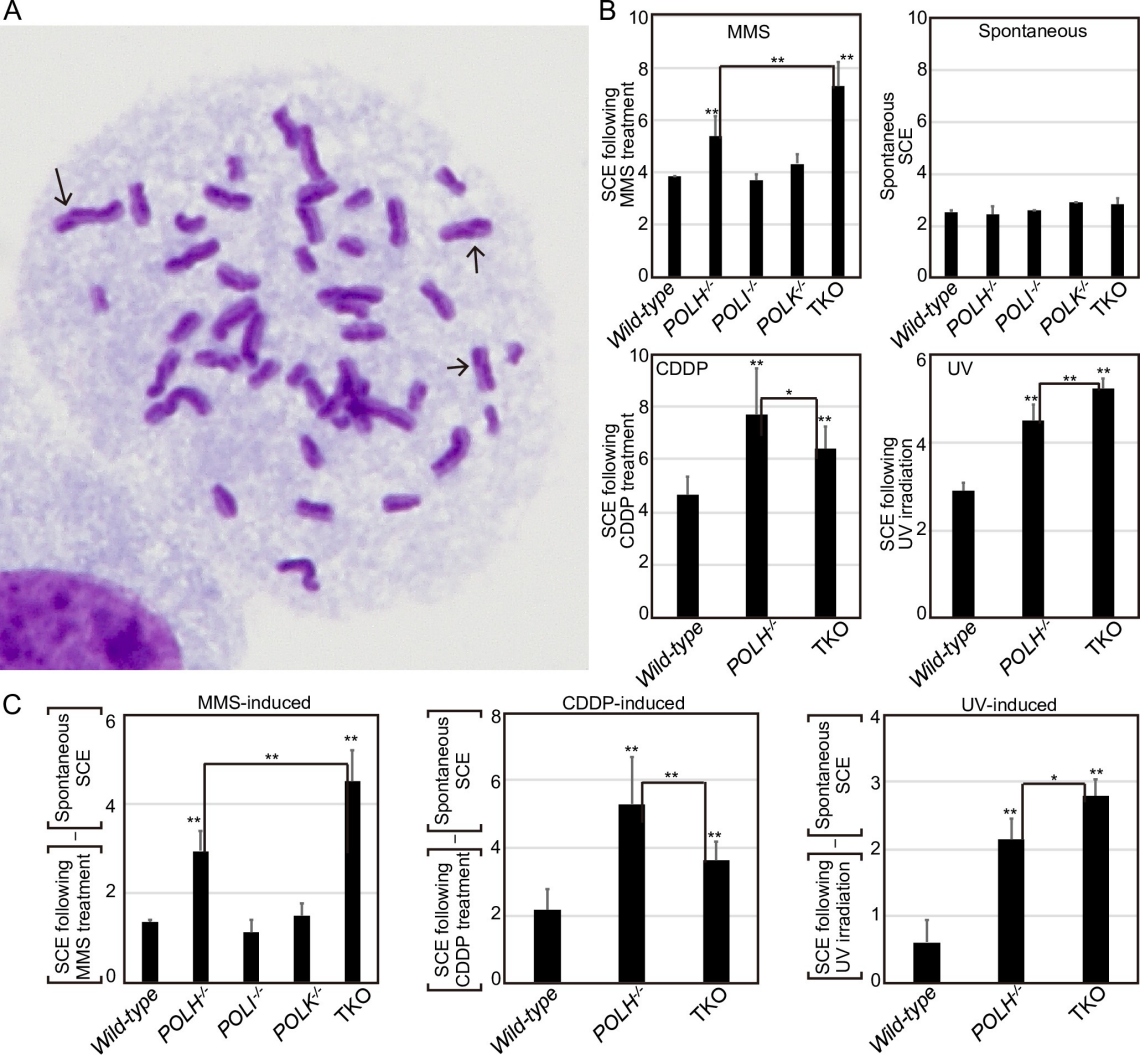
Increased number of SCEs due to TLS failure across DNA damage induced by MMS, CDDP, or UV. (A) Representative image showing SCEs in TK6 cells. (B) TK6 cells were continuously cultured in a medium containing BrdU (10 μM) and either CDDP (0.2 μM) or MMS (1 μg/mL) for 24 h. For UV irradiation, cells were exposed to UV (1 J/m^2^), then cultured in a medium containing BrdU for 24 h. Cells were treated with colcemid (0.1 μg/ml) to enrich for mitotic cells for the last 3 h of incubation. The number of spontaneous and induced SCEs in the macrochromosomes of 50 metaphase cells was scored at least two times. Error bars represent standard deviations from at least two independent analyses. Each data set is shown in S8 Fig. (C) The number of MMS-, CDDP-, or UV-induced SCEs was calculated by subtracting the number of spontaneous SCEs. Error bars represent standard deviations from at least two independent analyses. The *p*-value was calculated by Student’s *t*-test using all scored SCE data (*p < 0.05, **p < 0.01).

Given the complementary action of the three Y-family polymerases in TLS across MMS-induced base damage and the role of Polη in TLS across damage caused by CDDP and UV, we next asked if HR could compensate for TLS failure in the absence of Y-family polymerases. To this end, we analyzed SCEs to determine the efficiency of HR-mediated release from replication blockage (Fig 5A and 5B). Τhe level of spontaneous SCEs was slightly increased (~1.2 fold) in TKO cells, compared with *wild-type* cells, presumably because, as previously reported, lesions were more frequently channeled to HR in TLS-deficient cells [44, 50] (Fig 5B). We subtracted the number of spontaneous SCEs from the number of SCEs induced by the DNA-damaging agents to measure HR induced by replication blockages *via* MMS-, CDDP-, or UV-mediated DNA damage (Fig 5C). MMS-induced SCEs were slightly increased in *POLH*^*−/−*^, but not in *POLI*^*−/−*^ or *POLK*^*−/−*^ cells, while TKO cells exhibited a markedly increased level of SCEs (Fig 5C). These results suggest that the impact of reduced TLS ability in the absence of Polη was masked by HR-mediated release at damage induced by MMS. We next analyzed CDDP- or UV-induced SCEs in *wild-type*, *POLH*^*−/−*^, and TKO cells, since only *POLH*^*−/−*^, but not *POLI*^*−/−*^ or *POLK*^*−/−*^ cells, exhibited a markedly higher sensitivity and increased chromosomal aberrations after CDDP or UV treatment (Figs 1–3). The number of CDDP- or UV-induced SCEs was critically increased in *POLH*^*−/−*^, confirming the major role played by Polη in bypass replication across damage induced by CDDP or UV (Fig 5C). TKO cells showed significantly higher levels of UV-induced SCEs than did *POLH*^*−/−*^cells, confirming the role of Polι and Polκ as a backup for Polη in TLS across UV-induced damage. Strikingly, TKO cells showed reduced levels of CDDP-induced SCE, compared with *POLH*^*−/−*^cells. It is possible that the loss of Polι and Polκ in *POLH*^*−/−*^cells somehow interferes with HR, when replication stalls at CDDP-induced damage in the absence of Polη.

## Discussion

In this study, we investigated the relative contributions of the Y-family polymerases Polη, Polι, and Polκ in bypass replication of three distinct types of DNA damage. To this end, we disrupted *POLH*, *POLI*, and *POLK* genes in comprehensive combinations and generated single, double, and triple mutant cells from human TK6 cells. To determine the division of labor among the three Y-family polymerases, we examined cellular sensitivity to DNA-damaging agents (Fig 1, S4 Fig) and chromosomal aberrations induced by MMS, CDDP, and UV (Fig 2), with the following results. First, these three polymerases play complementary roles in bypass replication of templates carrying damages induced by MMS. Second, Polη plays the dominant role in TLS across CDDP-induced DNA damage, with the other polymerases contributing to this repair as a backup for Polη. Third, the tolerance of UV induced DNA lesions by Polι depends on its collaboration with Polη, and this collaborative activity represents only fraction of TLS performed by Polη itself.

We have demonstrated that Polη plays a dominant role in the bypass replication of UV-induced damage by partly collaborating with Polι (Figs 1 and 2). This epistatic relationship between *POLH* and *POLI* is consistent with the previously identified physical interaction between Polη and Polι and their tightly coordinated localization within the nucleus after UV irradiation [21]. Previously, involvement of Polι in the TLS of UV-induced damage as a backup for Polη was reported in human Burkitt’s lymphoma BL2 cell line [28]. The epistatic relationship between *POLH* and *POLI* demonstrated in this study is contrary to this previous result. This difference might be attributable to the deficient p53 pathway in Burkitt’s lymphoma BL2 cell line [51]. Another possible explanation for this difference may center on the different expressions of Y-family polymerases due to the deregulated expressions of Polι, and Polκ in each cancer cell lines [52]. The question is, how do Polη and Polι carry out bypass replication of UV-induced damage? TLS polymerases might compete with each other to perform bypass replication at the stalled replication fork at UV-induced damage; the Polη (partly complexed with Polι) might first address this lesion, with Polη playing a vital role in accurate TLS across CPDs [17, 53]. In the absence of the Polη, Polκ might serve as a critical backup, as evidenced by the observation that the TKO cells were significantly more sensitive to UV than were the *POLH*^*−/−*^/*POLI*^*−/−*^ cells (Figs 1 and 2). Collectively, these data suggest that all three Y-family polymerases (Polη, Polι, and Polκ) respond to UV damage, but the Polη-Polι complex might insert accurate nucleotides ApA opposite UV-mediated T-T dimers [17, 54], and Polκ-Polι may play a role in the absence of Polη. This model is consistent with a study demonstrating that Polζ and Polκ-Polι perform cooperative mutagenic TLS across T-T photodimers in cells in XP-V patients [55]. The other explanation for TKO’s enhanced cellular sensitivity to UV is that Polκ contributes to nucleotide-excision repair [49] to support cellular sensitivity independently from Polη and Polι, as evidenced by the observation that *POLH*^*−/−*^ and TKO cells showed indistinguishable fork-progression defects after 4-NQO treatment (Fig 4). This study showed that augmented chromosome aberrations in *POLH*^*−/−*^ cells in comparison to *wild-type* cells. Augmentation of chromosome aberrations upon UV damage has been also reported in mouse cells [56]. However, Federico *et al*. reported that si-RNA mediated depletion of Polη in U2OS cells causes cell death without showing augmentation of chromosome aberrations upon UV damage [57]. It is possible that these differences are caused by the difference of cell type (MEF, U2OS and TK6) and condition of experiment (*eg*. si-RNA depletion and knock-out, and incubation period after UV irradiation).

We here demonstrated that all single mutants (*POLH*^*−/−*^, *POLI*^*−/−*^, and *POLK*^*−/−*^) exhibit no significant defects in cellular tolerance to MMS and bypass replication across MMS induced damages in DNA fiber assay. However, *POLH*^*-/-*^ cells exhibited augmented number of MMS induced SCEs. It is possible that TLS at fork detected in the DNA fiber assay is preserved in *POLH*^*-/-*^ cells but post-replicative gap filling *via* TLS is reduced in this mutant cells and such defects are compensated by HR mediated repair.

The finding that cisplatin induces more SCE in *POLH*^*−/−*^ than in TKO, suggests that Polκ and/or Polι can somehow stimulate recombinogenic effects in response to this agent in the absence of Polη. This possibility is supported by the previous observations that Polι along with p53 is involved in the replication fork maintenance and restart via recombinogenic fork reversal [58].

Rev1 is also structurally Y-family polymerase, and this enzyme has deoxycytidyl transferase activity but lacks polymerase activity [59]. Rev1 physically and functionally associates with Polζ consisting of catalytic subunit Rev3 and non-catalytic subunits Rev7, PolD2 and PolD3 [60–62]. Epistatic relationship among *REV1*, *REV 3*, and *REV 7* in chicken DT40 cells well supports the functional cooperativity of these proteins [63]. In chicken DT40 cell, loss of Polη suppresses pronounced TLS defects in *REV3*^-/-^ cells [45], suggesting that Polη is also functionally linked with Polζ mediated TLS mechanism. Important questions for future studies concern a relationship between Polζ (consisting of Rev3-Rev7-Pold2-Pold3) and Y-family polymerases in human cells.

TLS contributes to chemoresistance of cancer cells, and some cancer cells show a resistant phenotype to anti-cancer drugs, including CDDP and the base alkylating agent temozolomide, owing to the overexpression of TLS polymerases. Thus, targeting TLS activity is an attractive method to improve cancer chemotherapy. Further understanding of the regulation of the TLS system, including Y-family DNA polymerases and other polymerases such as Polζ, might open up a novel avenue for future cancer chemotherapy based on genomic information and the transcriptome of cancer cells from each patient.

## Supporting information

S1 Fig Disruption of the *POLI* and *POLK* genes in human TK6 cells. (A, B) Schematic of a part of the *hPOLI* (A) or *hPOLK* (B) locus. Knockout constructs are shown below the locus. The filled boxes represent exons. The horizontal lines show the genomic region amplified for the targeting-vector arms. The indicated gRNA sequence was inserted into the BbsI site of pX330 (Cat# 42230, Addgene, US). pX330 expresses gRNA under the control of the U6 promoter and Cas9 under the chicken β-actin promoter. pX330-gRNA and the two indicated targeting vectors were transfected into TK6 cells using the Neon Transfection System (Thermo Fisher Scientific, PA). At 48 h after the transfection, appropriate selection reagents were added to select cells carrying maker genes. Target integrations of selection maker genes were confirmed by PCR using primers indicated by arrows. (C) The MIT specificity scores for each gRNA were calculated according to the method by Haeussler et al [39].
(PDF)

S2 Fig Confirmation of gene disruption of the *POLH*, *POLI*, and *POLK* genes in human TK6 cells. **(A)** TK6 cells with the indicated genotypes were subjected to RT-PCR using *POLH*-, *POLI-*, *POLK-*, or β-actin- (loading control) specific primers. Depletion of *POLH*, *POLI*, or *POLK* mRNA in the indicated cells was confirmed by RT-PCR. (B) TK6 cells with the indicated genotypes were subjected to western blot analysis using α-Polη, α-Polι, α-Polκ, and α-β-actin (loading control) specific antibodies. Loss of Polη, Polι, or Polκ protein in the indicated cells was confirmed by western blot analysis.
(PDF)

S3 Fig Y-family polymerases Polη, Polι, and Polκ are dispensable for cellular proliferation. (A) Relative growth rate plotted for the indicated genotypes. (B) Representative cell-cycle distribution for the indicated genotypes. DNA contents (stained by propidium iodide) are displayed on the x-axis on a linear scale, and the BrdU uptakes (stained by anti-BrdU antibody) are displayed on the y-axis on a logarithmic scale. The upper, lower left, and lower right gates correspond to cells in the S, G1, and G2/M phases, respectively. Red numbers show the percentage of cells that fall within each gate.
(PDF)

S4 Fig Polκ plays roles as a backup for Polη-Polι in the cellular tolerance to UV. TK6 cells were assessed for sensitivity to UV. Cell viability was assessed by colony survival assay, as described in the Materials and Methods. The dose of the indicated DNA-damaging agent is displayed on the x-axis on a linear scale, while the percentage of cell survival is displayed on the y-axis on a logarithmic scale. Error bars represent the standard deviation from three independent measurements.
(PDF)

S5 Fig Increased cellular sensitivity of TKO cells to MMS, CDDP, and UV in comparison to *wild-type* cells. (PDF)

S6 Fig Contribution of Polη, Polι, and Polκ to the prevention of chromosomal breakage after exposure to MMS, CDDP, and UV. The indicated TK6 cells were continuously cultured in medium containing CDDP (0.8 μM) or MMS (5 μg/mL) for 12 h or exposed to UV (4 J/m^2^) and cultured further for 12 h. Cells were treated with colcemid for the last 3 h. The number of chromosomal aberrations per 100 mitotic cells before and 12 h after treatment was scored three times; the average from three experiments is shown in the histogram on the y-axis. Error bars show the standard deviation from three independent experiments. The *p*-value was calculated by Student’s *t*-test (*p < 0.05).
(PDF)

S7 Fig Relationship between Polη, Polι, and Polκ in replication-fork progression after exposure to MMS-, CDDP-, or 4NQO-damaged DNA. The lengths of the CldU and IdU tracts were measured, and the CldU/IdU ratio for each replication fork was calculated for at least 100 replication forks. The histogram show the distribution of CldU/IdU ratios for replication forks in cells exposed to indicated DNA damaging agents. Indicated cells were incubated in medium containing CldU (25 μM) for 15 min, then incubated in medium containing IdU (250 μM) with indicated DNA damaging agents for 15 min. The CldU/IdU ratios are shown on the x-axis. The number of fibers in each section is shown on the y-axis. 100 forks from each cell line were analyzed. Median and standard error from at least 100 replication forks were indicated.
(PDF)

S8 Fig Increased number of SCEs due to TLS failure across DNA damage induced by MMS, CDDP, or UV. Histograms show the frequency of cells with the indicated number of SCEs per cell. SCE events in the macro-chromosomes of 50 metaphase cells were counted. Data in parenthesis represent the mean ± standard error.
(PDF)

S9 Fig Uncropped images. (PDF)
