# Supplementary material for: Division of labor of Y-family polymerases in translesion-DNA synthesis for distinct types of DNA damage
Source: PLoS One. 2021 Jun 1;16(6):e0252587. doi: 10.1371/journal.pone.0252587 (PMC8168857; doi:10.1371/journal.pone.0252587)
Supplement: S1 Fig — (A, B) Schematic of a part of the hPOLI (A) or hPOLK (B) locus. Knockout constructs are shown below the locus. The filled boxes represent exons. The horizontal lines show the genomic region amplified for the targeting-vector arms. The indicated gRNA sequence was inserted into the BbsI site of pX330 (Cat# 42230, Addgene, US). pX330 expresses gRNA under the control of the U6 promoter and Cas9 under the chicken β-actin promoter. pX330-gRNA and the two indicated targeting vectors were transfected into TK6 cells using the Neon Transfection System (Thermo Fisher Scientific, PA). At 48 h after the transfection, appropriate selection reagents were added to select cells carrying maker genes. Target integrations of selection maker genes were confirmed by PCR using primers indicated by arrows. (C) The MIT specificity scores for each gRNA were calculated according to the method by Haeussler et al [39]. (PDF) [file pone.0252587.s001.pdf]

A

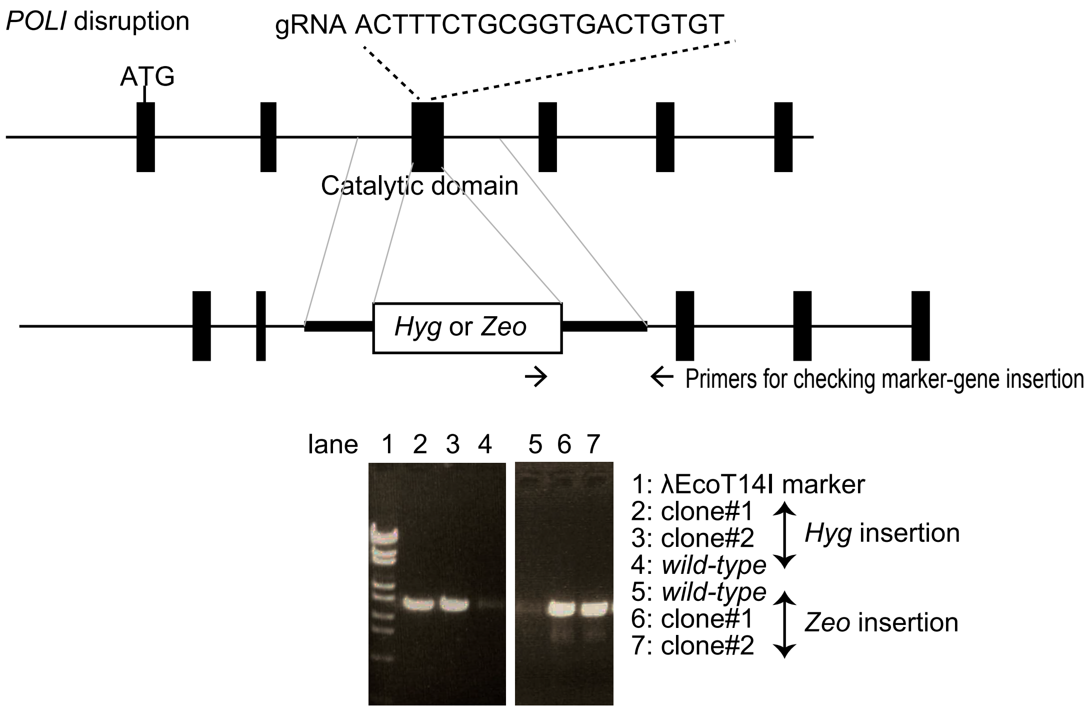

B

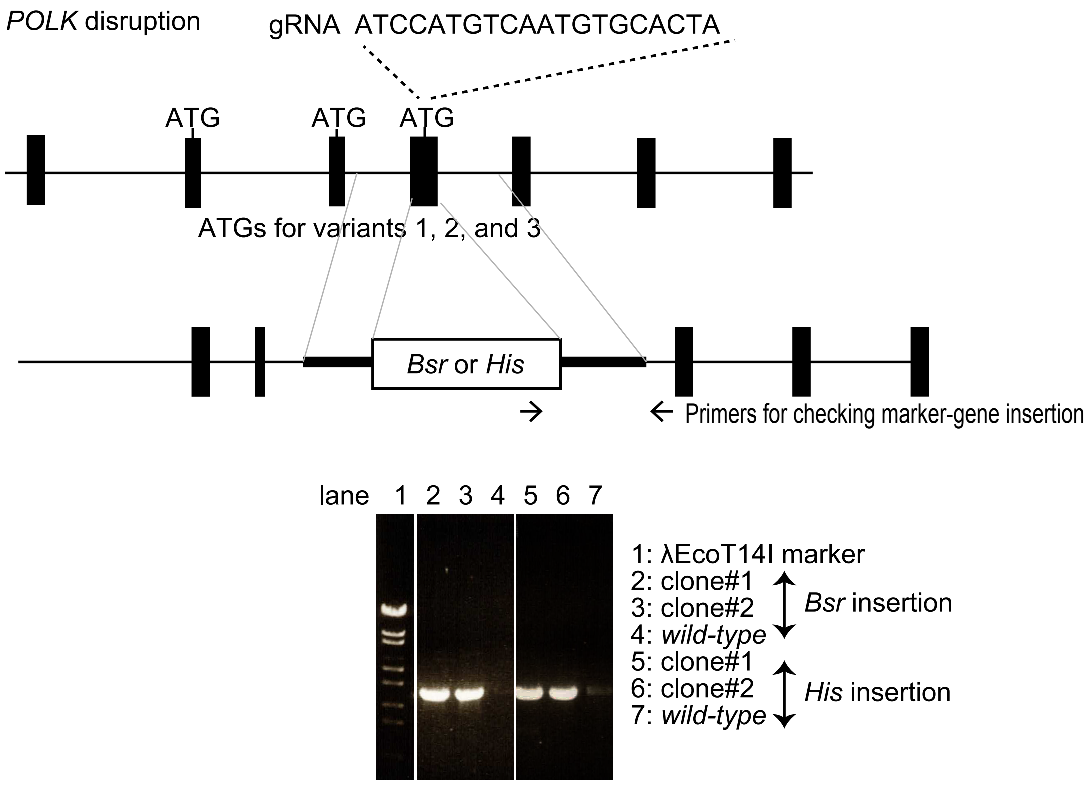

C

Gene	gRNA sequence	MIT Specificity Score
POLI	ACTTTCTGCGGTGACTGTGT	78
POLK	ATCCATGTCAATGTGCACTA	68
