# Supplementary figures and images for: Division of labor of Y-family polymerases in translesion-DNA synthesis for distinct types of DNA damage
Source: PLoS One. 2021 Jun 1;16(6):e0252587. doi: 10.1371/journal.pone.0252587 (PMC8168857; doi:10.1371/journal.pone.0252587)

A

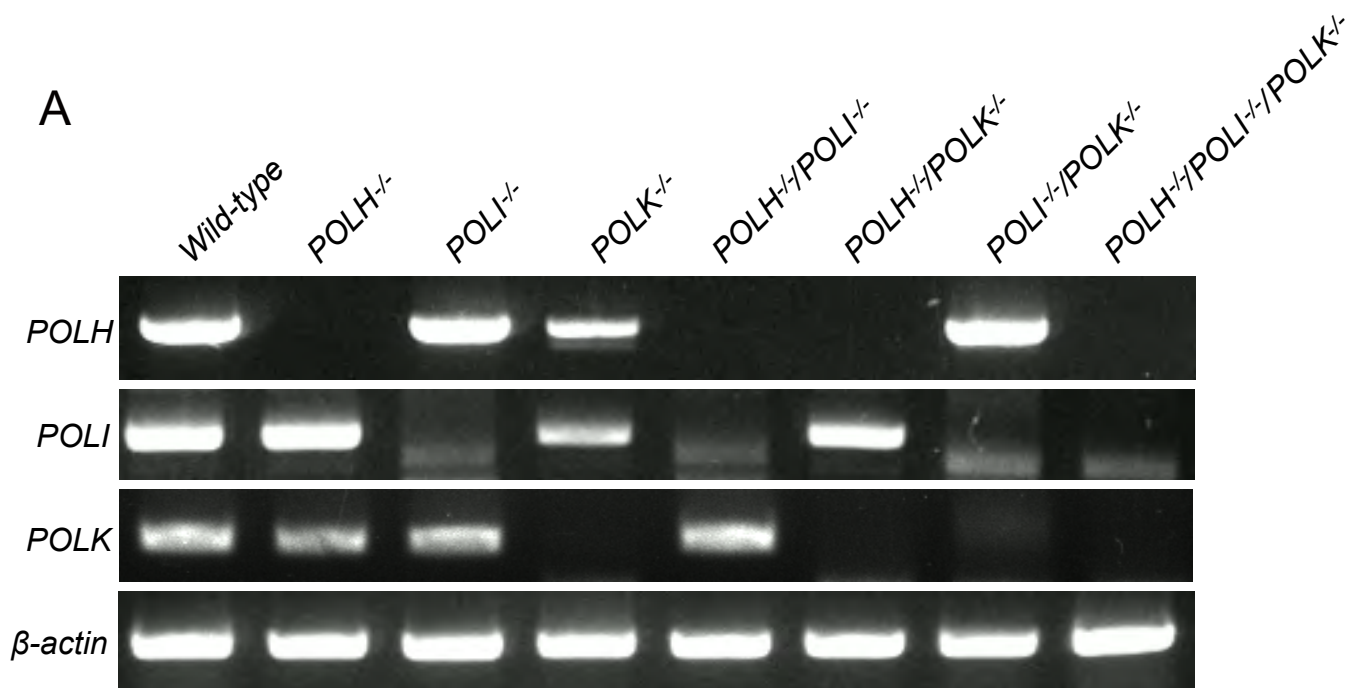

B

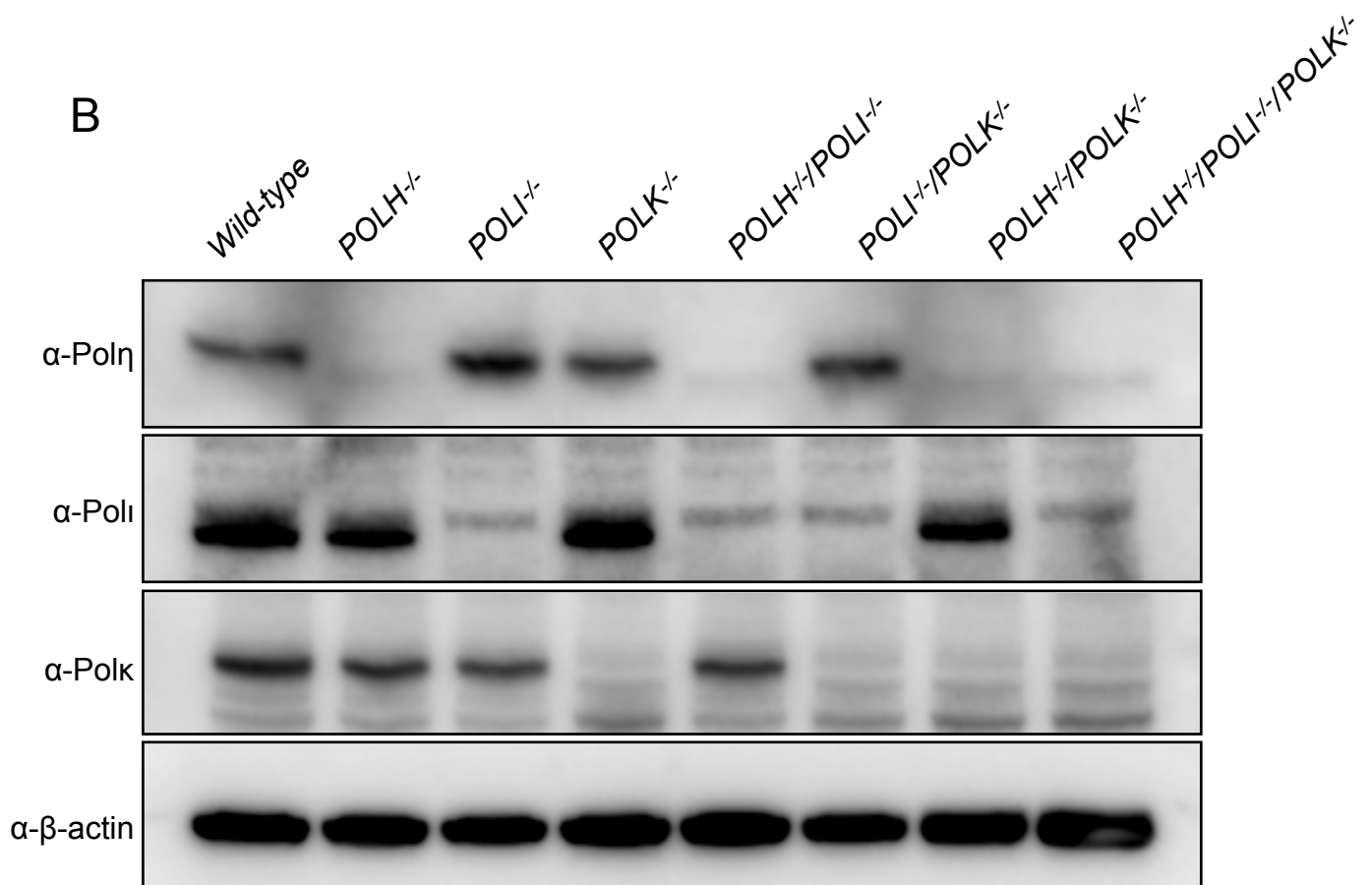

Supplement: S2 Fig — (A) TK6 cells with the indicated genotypes were subjected to RT-PCR using POLH-, POLI-, POLK-, or β-actin- (loading control) specific primers. Depletion of POLH, POLI, or POLK mRNA in the indicated cells was confirmed by RT-PCR. (B) TK6 cells with the indicated genotypes were subjected to western blot analysis using α-Polη, α-Polι, α-Polκ, and α-β-actin (loading control) specific antibodies. Loss of Polη, Polι, or Polκ protein in the indicated cells was confirmed by western blot analysis. (PDF) [file pone.0252587.s002.pdf]

A

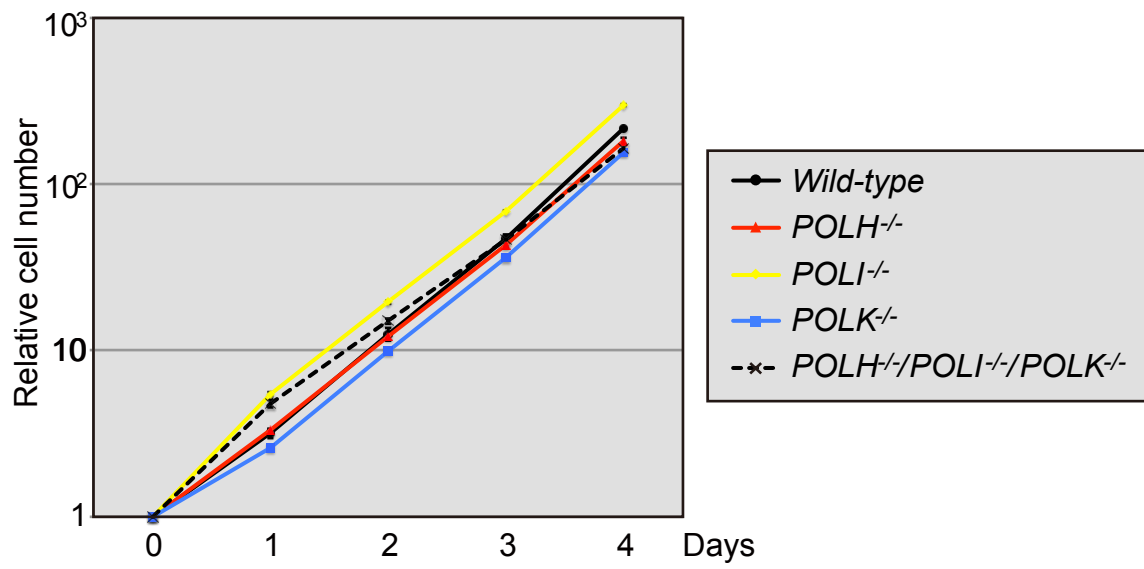

B

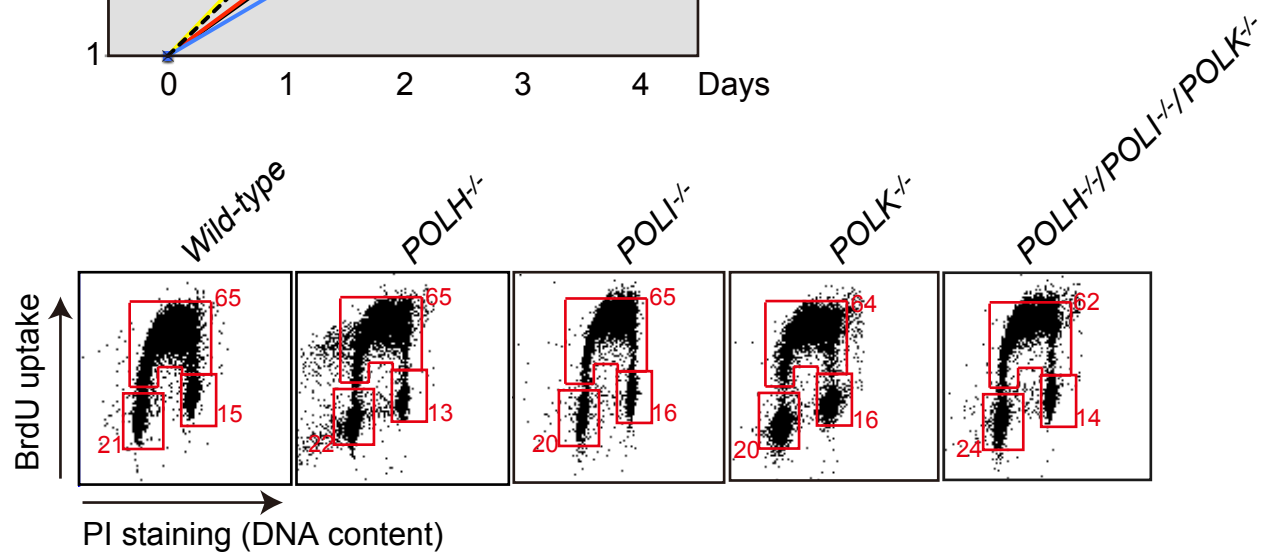

Supplement: S3 Fig — (A) Relative growth rate plotted for the indicated genotypes. (B) Representative cell-cycle distribution for the indicated genotypes. DNA contents (stained by propidium iodide) are displayed on the x-axis on a linear scale, and the BrdU uptakes (stained by anti-BrdU antibody) are displayed on the y-axis on a logarithmic scale. The upper, lower left, and lower right gates correspond to cells in the S, G1, and G2/M phases, respectively. Red numbers show the percentage of cells that fall within each gate. (PDF) [file pone.0252587.s003.pdf]

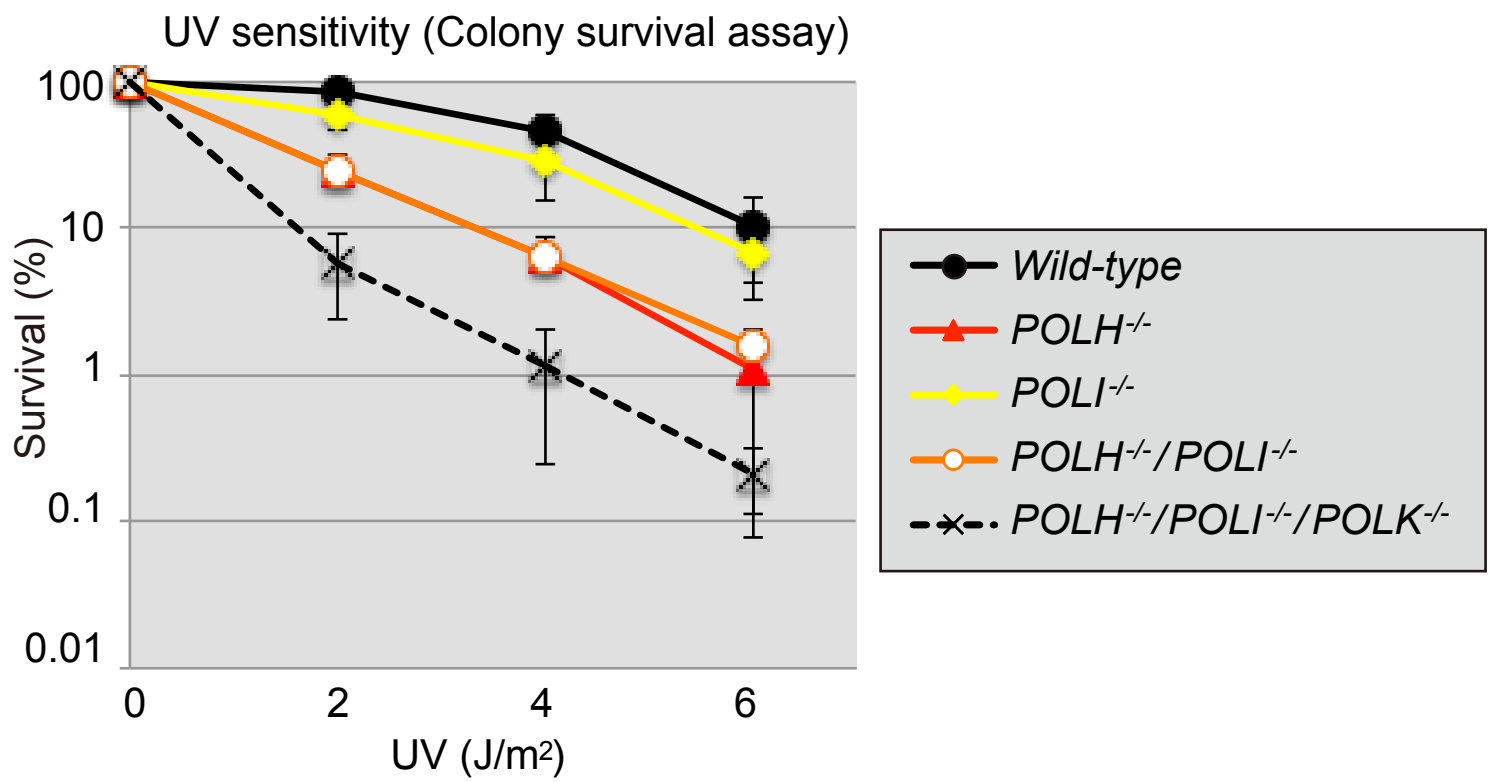

Supplement: S4 Fig — TK6 cells were assessed for sensitivity to UV. Cell viability was assessed by colony survival assay, as described in the Materials and Methods. The dose of the indicated DNA-damaging agent is displayed on the x-axis on a linear scale, while the percentage of cell survival is displayed on the y-axis on a logarithmic scale. Error bars represent the standard deviation from three independent measurements. (PDF) [file pone.0252587.s004.pdf]

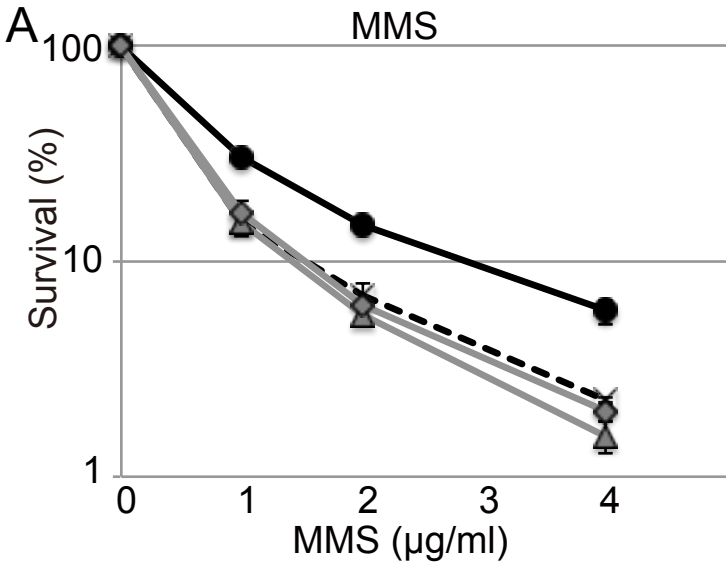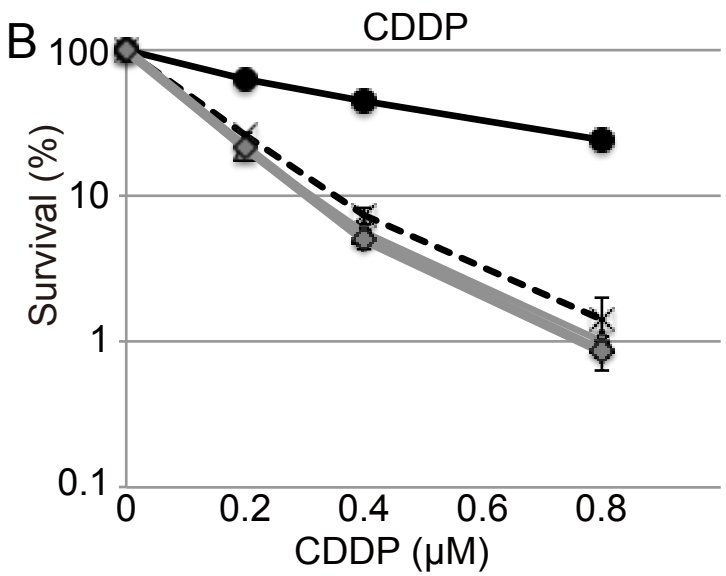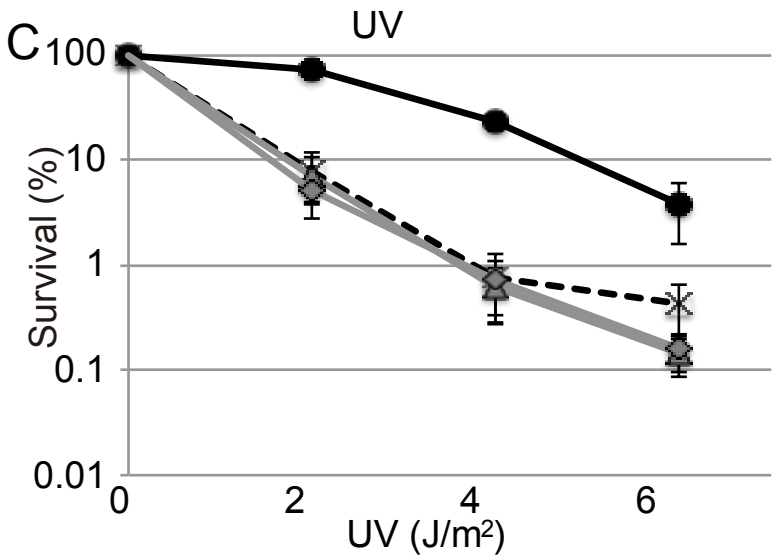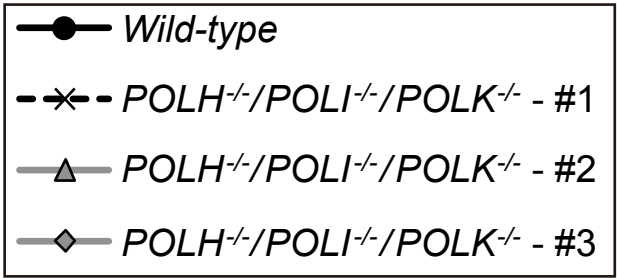

Supplement: S5 Fig — (PDF) [file pone.0252587.s005.pdf]

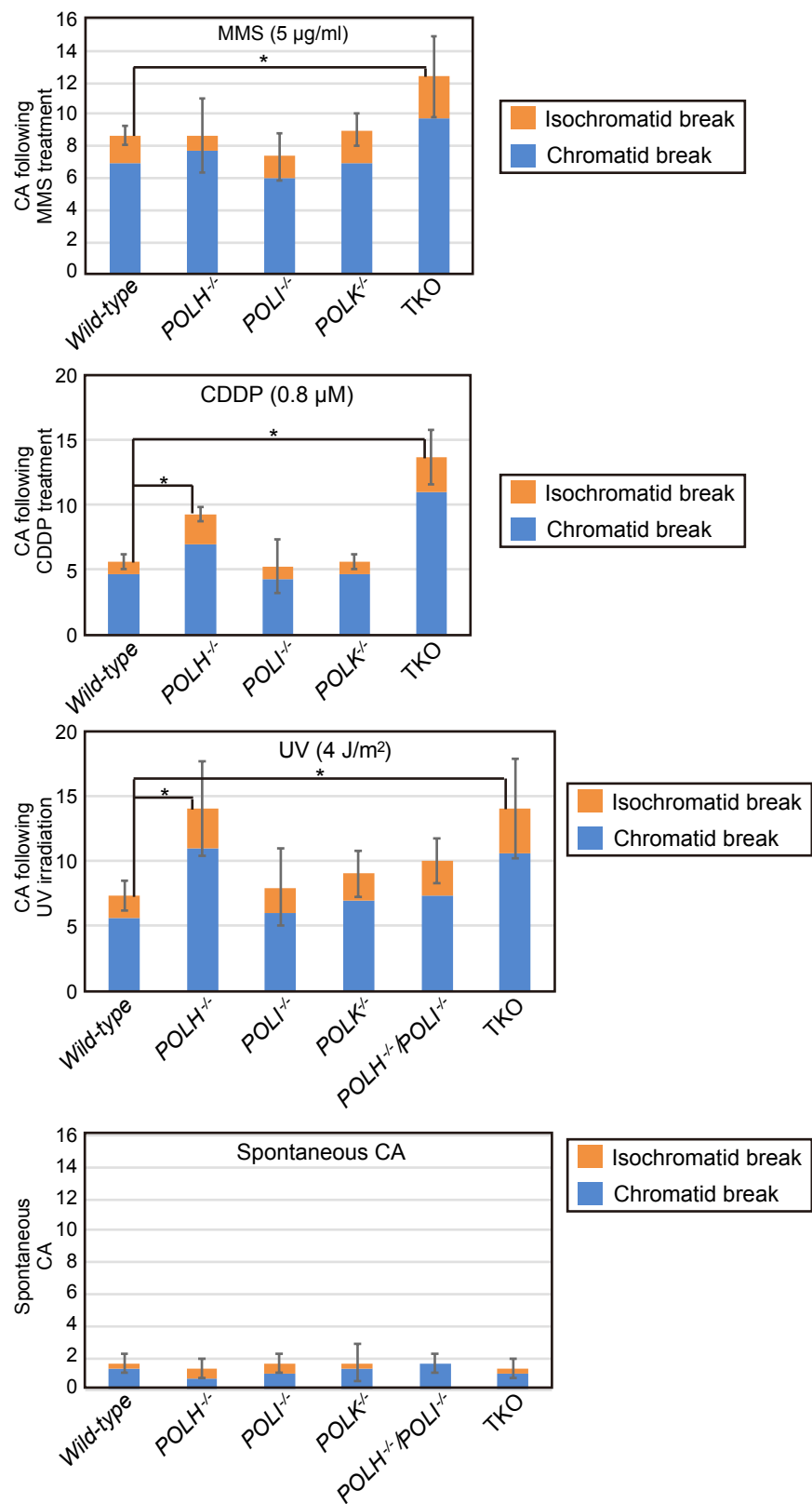

Supplement: S6 Fig — The indicated TK6 cells were continuously cultured in medium containing CDDP (0.8 μM) or MMS (5 μg/mL) for 12 h or exposed to UV (4 J/m2) and cultured further for 12 h. Cells were treated with colcemid for the last 3 h. The number of chromosomal aberrations per 100 mitotic cells before and 12 h after treatment was scored three times; the average from three experiments is shown in the histogram on the y-axis. Error bars show the standard deviation from three independent experiments. The p-value was calculated by Student’s t-test (*p < 0.05). (PDF) [file pone.0252587.s006.pdf]

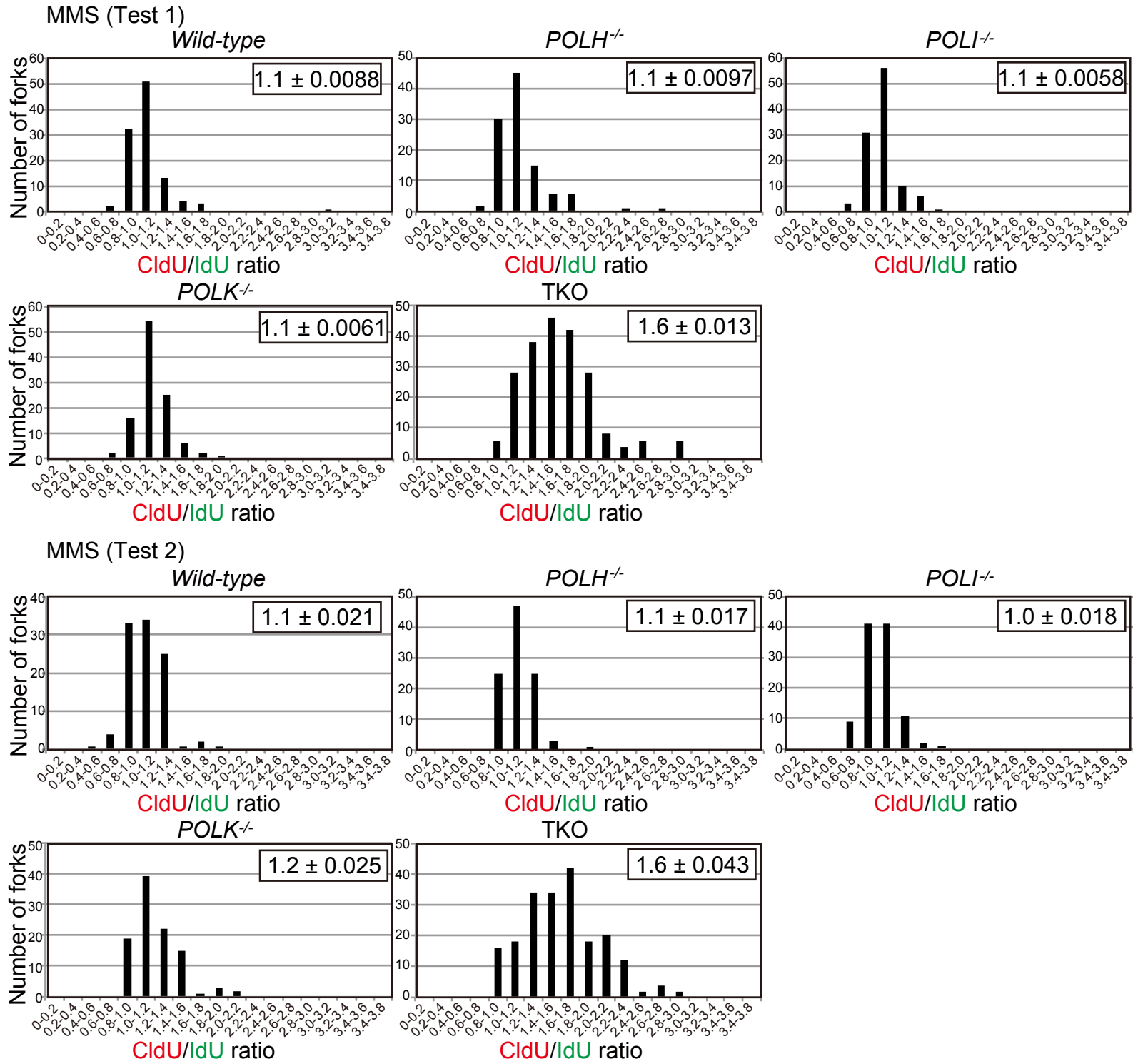

MMS (Test 3)

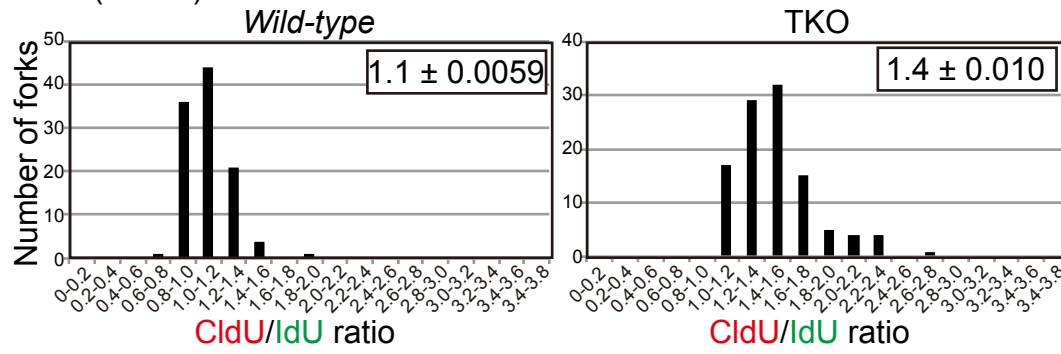

MMS (Test4)

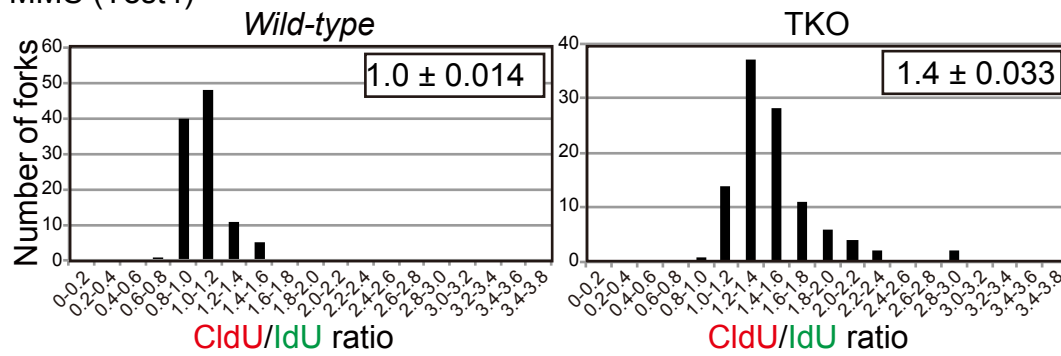

CDDP (Test 1)

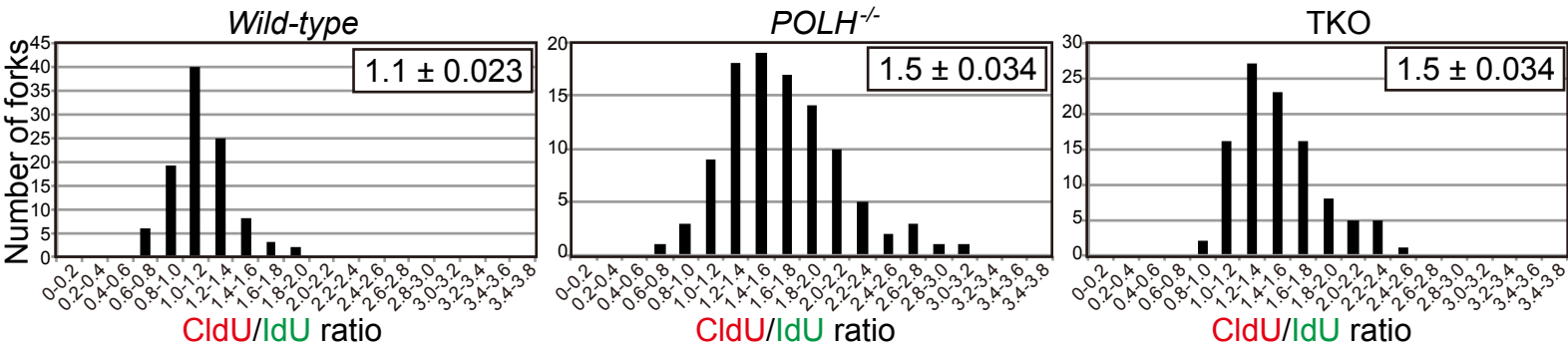

CDDP (Test 2)

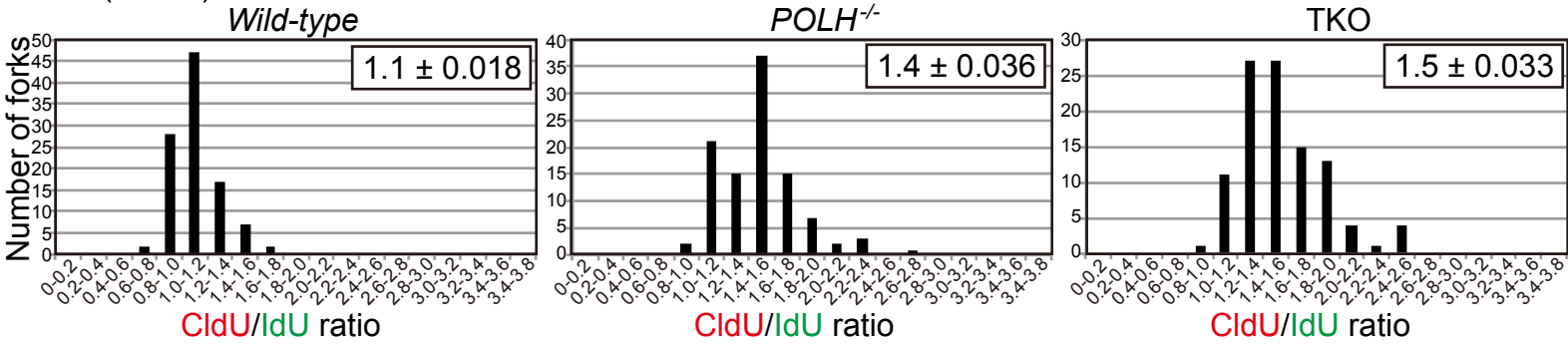

4NQO (Test 1)

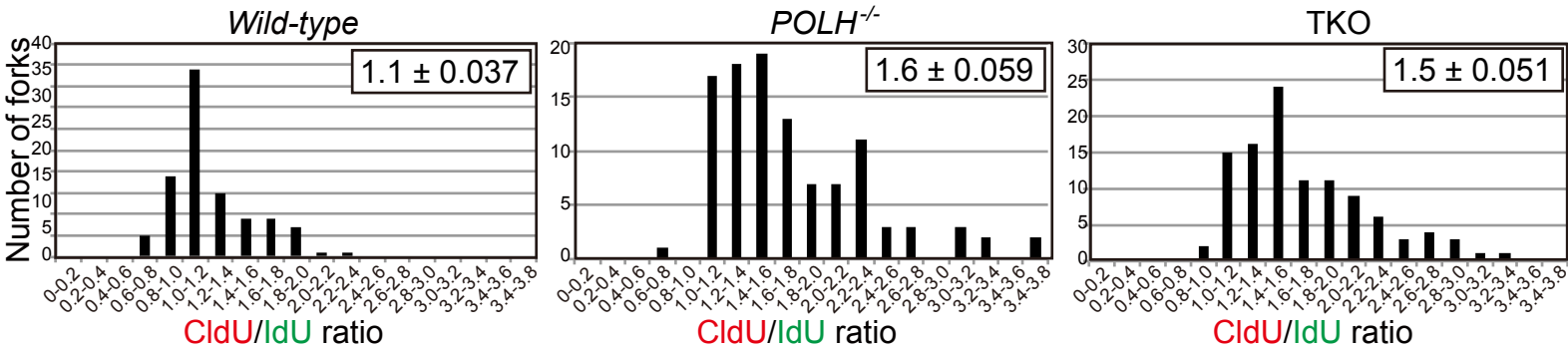

4NQO (Test 2)

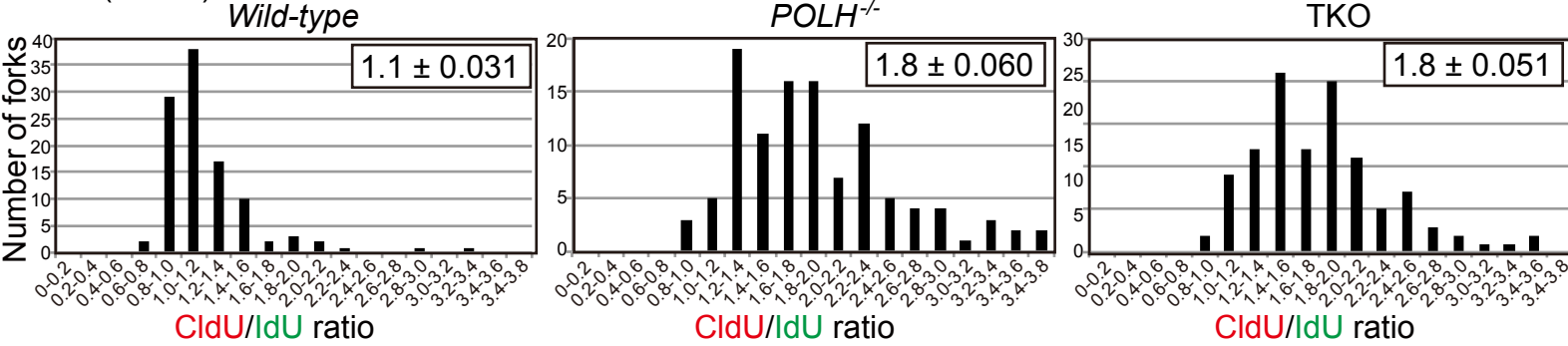

Supplement: S7 Fig — The lengths of the CldU and IdU tracts were measured, and the CldU/IdU ratio for each replication fork was calculated for at least 100 replication forks. The histogram show the distribution of CldU/IdU ratios for replication forks in cells exposed to indicated DNA damaging agents. Indicated cells were incubated in medium containing CldU (25 μM) for 15 min, then incubated in medium containing IdU (250 μM) with indicated DNA damaging agents for 15 min. The CldU/IdU ratios are shown on the x-axis. The number of fibers in each section is shown on the y-axis. 100 forks from each cell line were analyzed. Median and standard error from at least 100 replication forks were indicated. (PDF) [file pone.0252587.s007.pdf]
