# Supplementary material for: Division of labor of Y-family polymerases in translesion-DNA synthesis for distinct types of DNA damage
Source: PLoS One. 2021 Jun 1;16(6):e0252587. doi: 10.1371/journal.pone.0252587 (PMC8168857; doi:10.1371/journal.pone.0252587)

Inomata *et al*. Fig. S8_1

MMS (Test1)

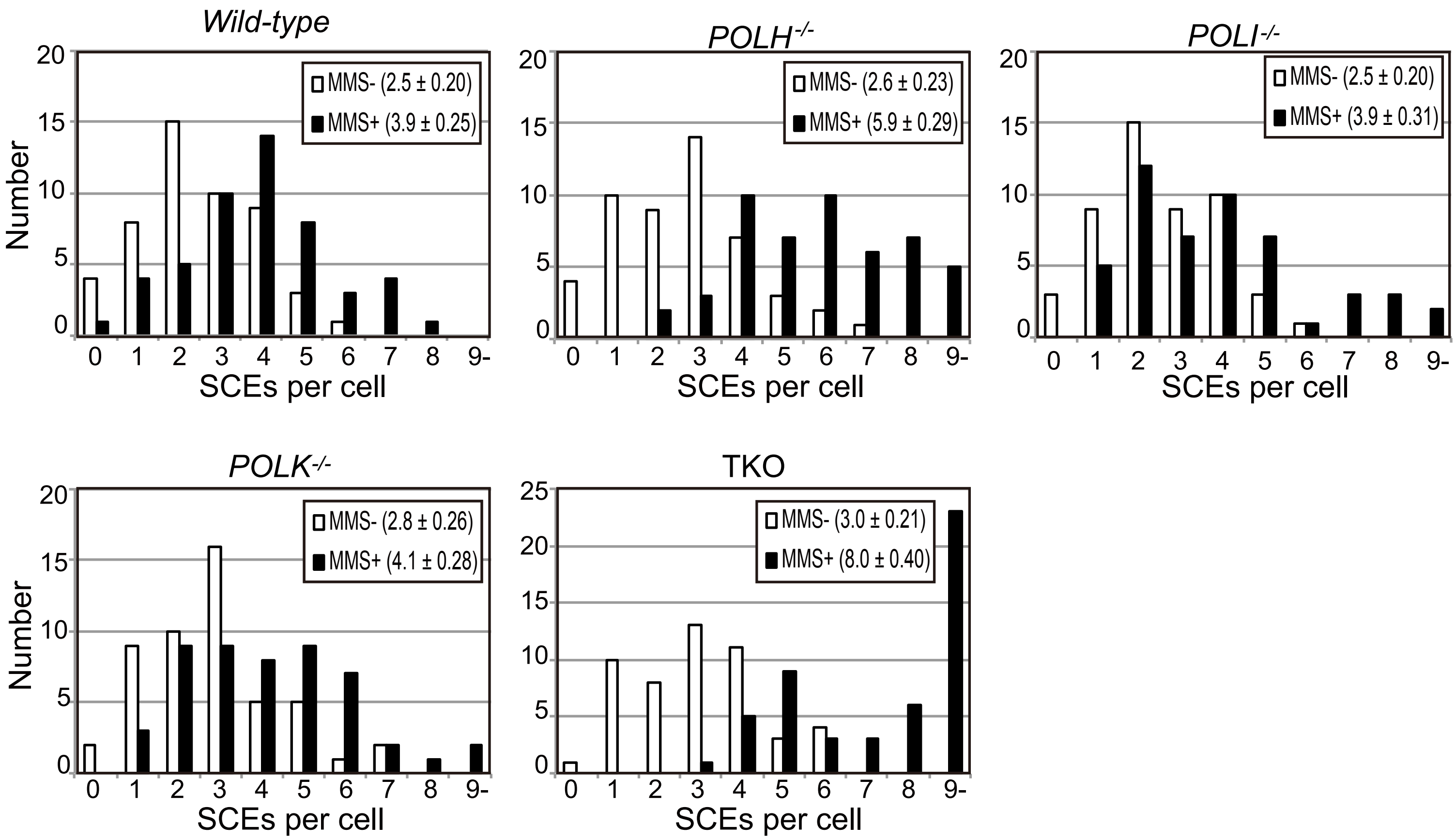

MMS (Test2)

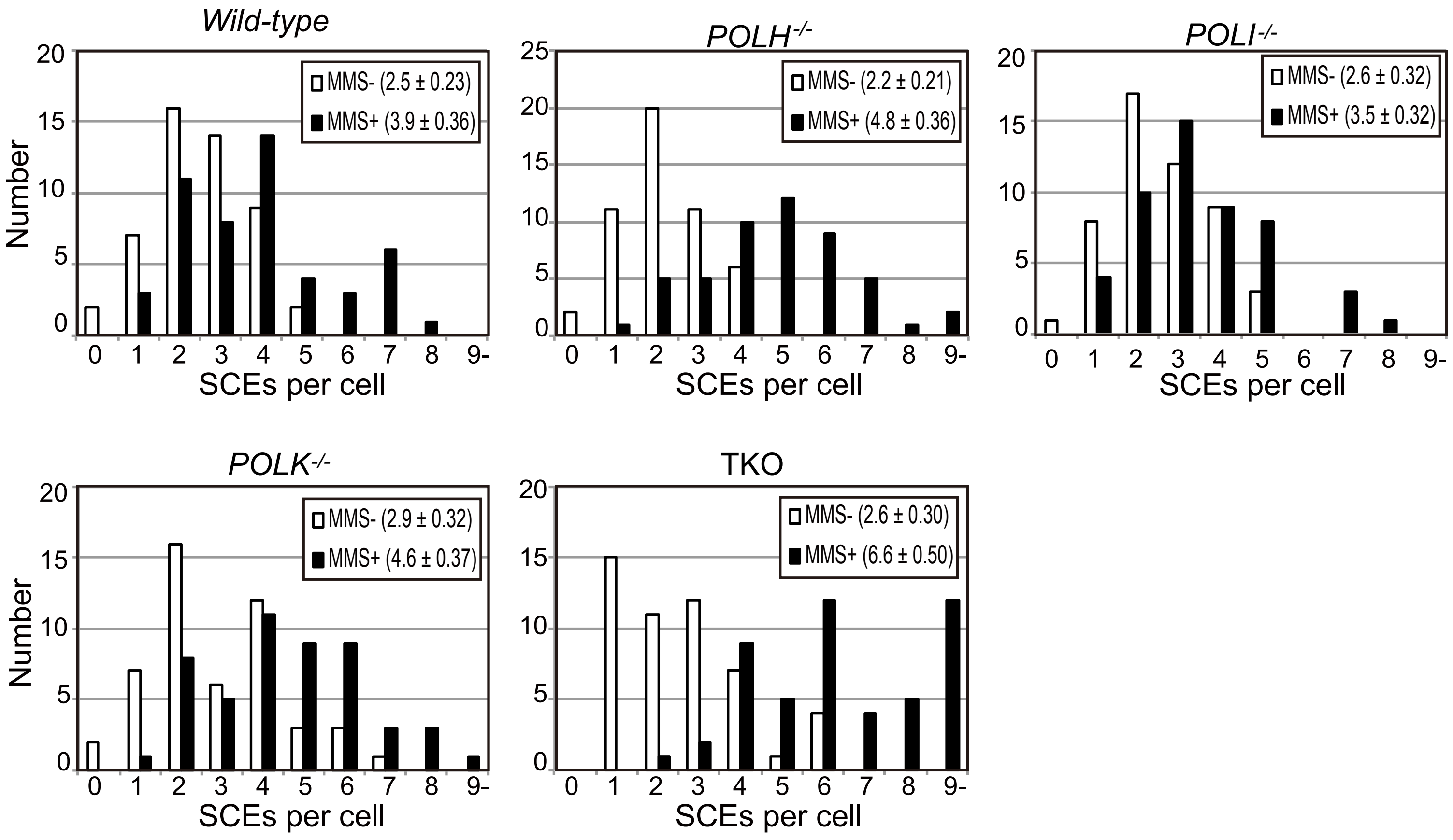

Inomata *et al*. Fig. S8_2

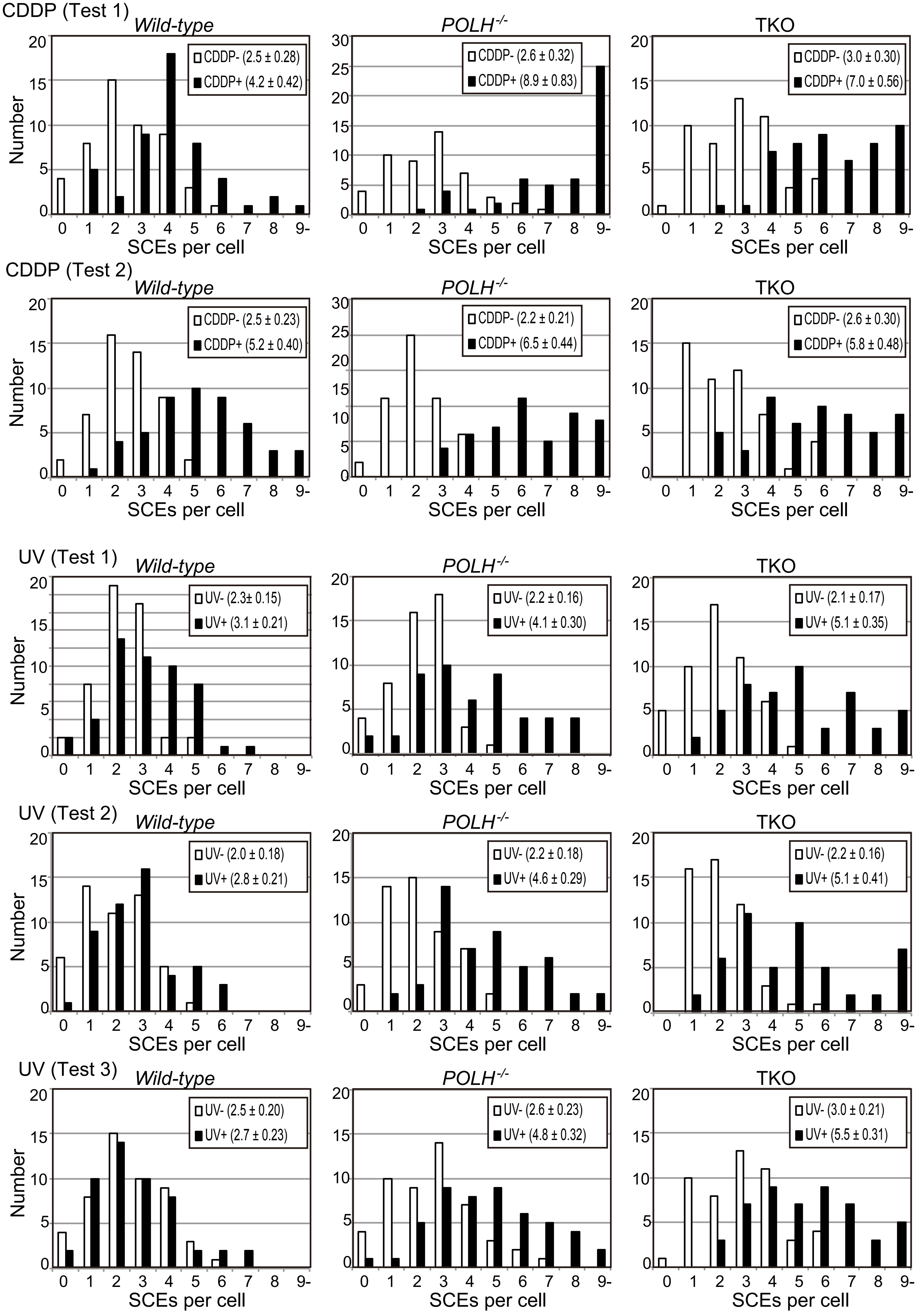

Supplement: S8 Fig — Histograms show the frequency of cells with the indicated number of SCEs per cell. SCE events in the macro-chromosomes of 50 metaphase cells were counted. Data in parenthesis represent the mean ± standard error. (PDF) [file pone.0252587.s008.pdf]
