# Supplementary material for: Division of labor of Y-family polymerases in translesion-DNA synthesis for distinct types of DNA damage
Source: PLoS One. 2021 Jun 1;16(6):e0252587. doi: 10.1371/journal.pone.0252587 (PMC8168857; doi:10.1371/journal.pone.0252587)

Inomata *et al.* Fig. S9

Uncropped blot used in Supplementary Fig. S1A

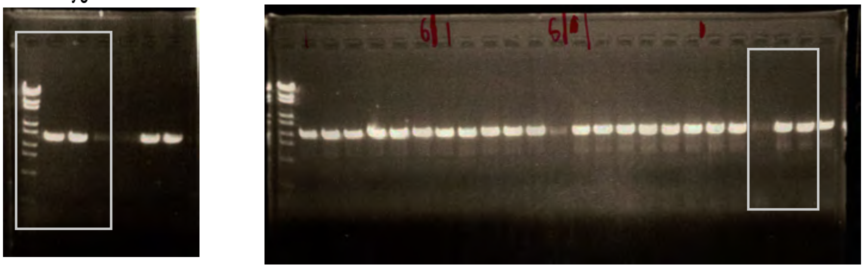

Uncropped blot used in Supplementary Fig. S1B

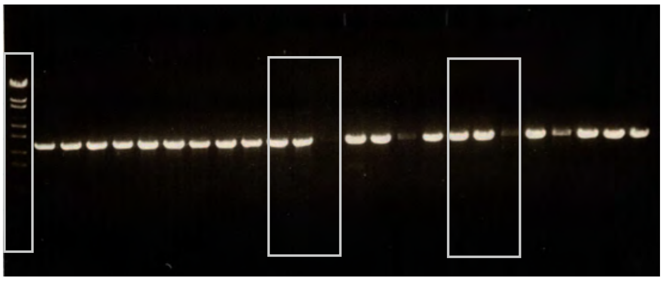

Uncropped blot used in Supplementary Fig. S2

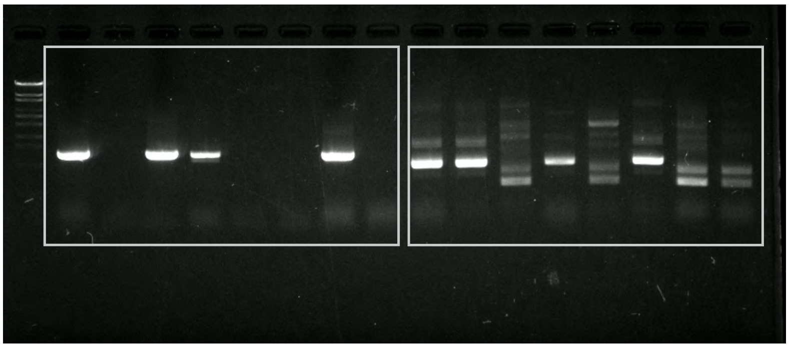

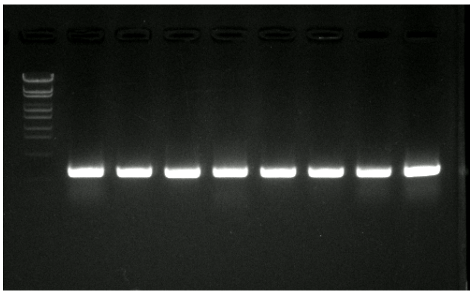

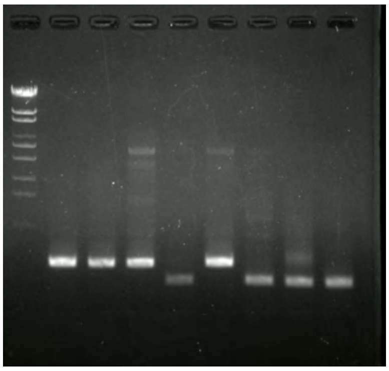

Supplement: S9 Fig — (PDF) [file pone.0252587.s009.pdf]
